# REDD1 functions at the crossroads between the therapeutic and adverse effects of topical glucocorticoids

**DOI:** 10.15252/emmm.201404601

**Published:** 2014-12-11

**Authors:** Gleb Baida, Pankaj Bhalla, Kirill Kirsanov, Ekaterina Lesovaya, Marianna Yakubovskaya, Kit Yuen, Shuchi Guo, Robert M Lavker, Ben Readhead, Joel T Dudley, Irina Budunova

**Affiliations:** 1 Department of Dermatology, Northwestern UniversityChicago, IL, USA; 2 N. Blokhin Cancer Research Center, RAMSMoscow, Russia; 3 Department of Genetics and Genomic Sciences, Icahn School of Medicine at Mount SinaiNew York, NY, USA

**Keywords:** glucocorticoid, glucocorticoid receptor, mTOR, REDD1, skin atrophy

## Abstract

Cutaneous atrophy is the major adverse effect of topical glucocorticoids; however, its molecular mechanisms are poorly understood. Here, we identify stress-inducible mTOR inhibitor REDD1 (regulated in development and DNA damage response 1) as a major molecular target of glucocorticoids, which mediates cutaneous atrophy. In REDD1 knockout (KO) mice, all skin compartments (epidermis, dermis, subcutaneous fat), epidermal stem, and progenitor cells were protected from atrophic effects of glucocorticoids. Moreover, REDD1 knockdown resulted in similar consequences in organotypic raft cultures of primary human keratinocytes. Expression profiling revealed that gene activation by glucocorticoids was strongly altered in REDD1 KO epidermis. In contrast, the down-regulation of genes involved in anti-inflammatory glucocorticoid response was strikingly similar in wild-type and REDD1 KO mice. Integrative bioinformatics analysis of our and published gene array data revealed similar changes of gene expression in epidermis and in muscle undergoing glucocorticoid-dependent and glucocorticoid-independent atrophy. Importantly, the lack of REDD1 did not diminish the anti-inflammatory effects of glucocorticoids in preclinical model. Our findings suggest that combining steroids with REDD1 inhibitors may yield a novel, safer glucocorticoid-based therapies.

## Introduction

Glucocorticoid hormones are essential regulators of proliferation, differentiation, and metabolism in skin. They are also effective anti-inflammatory drugs widely used to treat the hyperproliferative and inflammatory skin diseases such as atopic dermatitis and psoriasis (Schäcke *et al*, 2006; Schoepe *et al*, 2006). Unfortunately, their beneficial therapeutic effects are often accompanied by numerous adverse effects including skin atrophy, characterized by a profound loss in skin thickness and elasticity combined with decreased barrier function. Skin atrophy involves all skin compartments, that is, epidermis, dermis, sebaceous glands, and subcutaneous (s.c.) fat. Typical epidermal changes include a reduction in thickness, decreased number and size of keratinocytes, diminished stratum corneum and intercellular lipid lamella (Jablonska *et al*, 1979; Lehmann *et al*, 1983; Zheng *et al*, 1984; Lubach & Kietzmann, 1988; Schoepe *et al*, 2006). These changes are combined with an altered orientation and packing of collagen and elastin fibers, and decreased cellularity in the dermis (Lehmann *et al*, 1983; Schoepe *et al*, 2006). In addition, in mice topical glucocorticoids and in patients intradermally injected glucocorticoids induce drastic atrophy/lypolysis of s.c. fat (Woodbury & Kligman, 1992; Imagawa & Ohkuma, 2010). Although steroid-induced skin atrophy is well known and characterized morphologically, the underlying molecular mechanisms are poorly understood.

Glucocorticoids act via a specific receptor (the glucocorticoid receptor, GR), which is a ligand-dependent transcription factor (Adcock, 2001; Necela & Cidlowski, 2004; Vandevyver *et al*, 2012; Ratman *et al*, 2013). In the absence of glucocorticoids, GR resides in the cytoplasm in a complex with molecular chaperones that inhibit GR nuclear import. Upon ligand binding, GR undergoes phosphorylation, dimerization, and nuclear translocation. Gene transactivation (TA) by glucocorticoids requires binding of GR homodimers to palindromic glucocorticoid-responsive elements (GRE) in gene promoters. Transrepression (TR) by glucocorticoids is mediated by diverse mechanisms including interaction between GR and other transcription factors, such as major pro-inflammatory factors NF-κB and AP-1 (De Bosscher *et al*, 2003; Necela & Cidlowski, 2004; Schäcke *et al*, 2006; Chebotaev *et al*, 2007a; Ratman *et al*, 2013). TR by GR is critical for anti-inflammatory effects of glucocorticoids. In contrast, many metabolic side effects of oral steroids related to the maintenance of the *hypothalamic–pituitary–adrenal* axis, glucose metabolism, and osteoporosis are largely dependent on TA (Schäcke *et al*, 2006, 2009; De Bosscher *et al*, 2010; Nixon *et al*, 2013; Ratman *et al*, 2013).

We discovered recently that glucocorticoids induced a robust activation of REDD1 (regulated in development and DNA damage response) in mouse and human skin (Baida *et al*, 2013). REDD1 is a stress response gene induced by hypoxia, DNA damage, nutrient or energy deprivation, or by endoplasmic reticulum stress (Shoshani *et al*, 2002; Ellisen *et al*, 2002; Brugarolas *et al*, 2004; Lin *et al*, 2005; Sofer *et al*, 2005; Wang *et al*, 2006; DeYoung *et al*, 2008). REDD1 is also activated by glucocorticoids and is a direct GR target (Wang *et al*, 2006; Shimizu *et al*, 2011). It inhibits mammalian target of rapamycin (mTOR) by stabilizing the tuberous sclerosis protein 1 (TSC1)–TSC2 inhibitory complex (Brugarolas *et al*, 2004; Ellisen, 2005; Wang *et al*, 2006; DeYoung *et al*, 2008; Mata *et al*, 2011). mTOR regulation by REDD1 contributes to the control of cell growth and size in *Drosophila* and in mammals. These findings suggest that abnormalities of REDD1 signaling may disrupt energy homeostasis (Ellisen *et al*, 2002; Shoshani *et al*, 2002; Sofer *et al*, 2005; Katiyar *et al*, 2009).

REDD1 is involved in another atrophogenic effect of glucocorticoids, muscle waste (Wang *et al*, 2006; Shimizu *et al*, 2011). It is also well understood that cross-talk between anabolic mTOR and catabolic GR is important to maintain the mass of skeletal muscle (Shimizu *et al*, 2011). Such cross-talk, as well as the role of REDD1 in steroid-induced atrophy in the skin, has not been considered.

REDD1 KO mice (Brafman *et al*, 2004; Sofer *et al*, 2005) are resistant to a variety of pathological conditions caused by stress, such as oxidative stress in the retina, emphysema induced by tobacco smoke, and ceramide-induced apoptosis of the lung epithelial cells (Brafman *et al*, 2004; Yoshida *et al*, 2010; Kamocki *et al*, 2013). However, neither adverse nor therapeutic effects of glucocorticoids have been studied on REDD1 KO background.

Here, we used REDD1 KO mice to examine the role of REDD1 in skin. We report that REDD1 KO animals are resistant to glucocorticoid-induced skin atrophy compared to wild-type isogenic animals. Similarly, organotypic raft cultures of primary human keratinocytes after REDD1 knockdown were completely protected from the hypoplastic effects of glucocorticoids. In contrast, REDD1 was dispensable for the anti-inflammatory action of glucocorticoids. In agreement, the comparison of transcriptional response to glucocorticoids in the epidermis of wild-type and REDD1 KO mice revealed that REDD1 is critically important for the TA by the glucocorticoids, especially the genes related to lipid and protein metabolism/catabolism, but not for the TR of the genes related to glucocorticoid anti-inflammatory effects.

## Results

### Topical glucocorticoids induce skin atrophy and activate REDD1 expression

**Figure 1:**
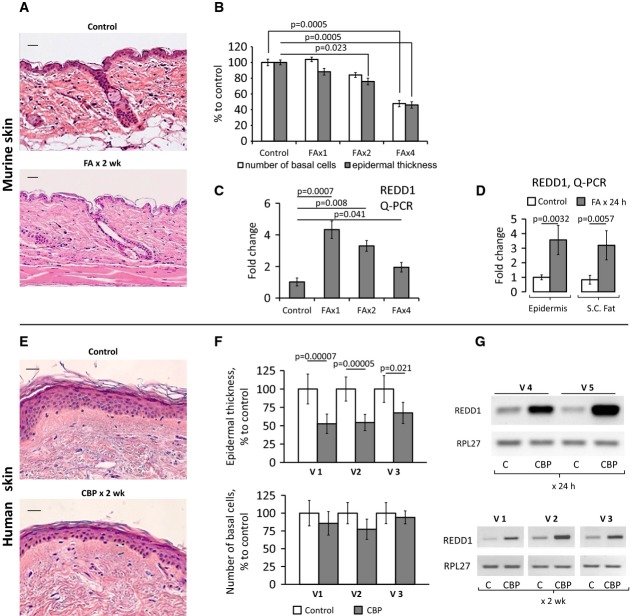
Topical glucocorticoids induce epidermal atrophy and REDD1 mRNA expression A–G B6D2 mice were treated topically with acetone (vehicle control) or glucocorticoid FA (2 μg/animal), every 72 h for 2 weeks. Human volunteers were treated with 0.05% CPB cream applied to the right arm skin once or daily for 2 weeks. Untreated skin from the left arm was used as a control. H&E staining of mouse skin (A) and human skin (E). Scale bars are 20 μm (A) and 40 μm (E). Morphometric analysis of epidermal thickness and number of basal keratinocytes in mouse skin treated with FA 1, 2, and 4 times (B), and human skin treated daily for 2 weeks (F). REDD1 mRNA expression in mouse epidermis 8 h after 1^st^, 2^nd^, and 4^th^ applications of FA (C, Q-PCR), in mouse epidermis and s.c. adipose, 24 h after FA (D, Q-PCR), and in human skin 24 h after single (G, volunteers V4 and V5) and 2-week (G, volunteers V1, V2, V3, RT–PCR) treatment with CBP. RPL27 was used as a cDNA normalization control. In human skin, the means ± SD were calculated in each individual sample compared to the untreated skin from the same individual (30 measurements/condition). In mouse skin, the means ± SD were calculated for three individual skin samples/condition in one representative experiment (30 measurements/condition) out of three experiments. Q-PCR results are the means ± SD calculated for three individual RNA samples/condition. Statistical analysis for differences between treatment and control was done by the unpaired two-tailed *t*-test.

We used clinically relevant glucocorticoid regiments known to induce significant skin atrophy in mice and in patients (Schoepe *et al*, 2006, 2010; Chebotaev *et al*, 2007b). B6D2 mice (F1 C57Bl/6 × DBA) that we used previously to study the side effects of glucocorticoids (Chebotaev *et al*, 2007b) were treated with medium potency glucocorticoid fluocinolone acetonide (FA, 2 μg/animal). For human volunteers, we used one of the most potent steroids, clobetasol propionate (CBP, 0.05% cream). In mice, a 2-week treatment caused a marked 50% epidermal thinning and 50% depletion of the interfollicular basal keratinocytes (Fig1A and B). A similar degree of epidermal hypoplasia was observed in humans after 2-week treatment; however, the negative effect on basal keratinocytes was less pronounced (Fig1E and F).

We recently reported that REDD1 was at the top of the list of genes up-regulated in mouse epidermis upon FA treatment (Baida *et al*, 2013). Using Q-PCR, we showed that REDD1 mRNA was increased by ∼4.5-fold in the epidermis of B6D2 mice, 4–24 h after the first FA application (Fig1C and D), and stayed significantly above the control level for the duration of treatment. REDD1 mRNA was also strongly up-regulated in human skin treated with CBP for 24 h–2 weeks (Fig1G).

Because the atrophic changes in mouse epidermis due to FA treatment were paralleled by the complete depletion of s.c. adipose tissue (Fig1A), we assessed REDD1 in adipose. The purity of s.c. adipose isolation was verified using specific adipocyte and keratinocyte markers (Supplementary Fig S1). We found that REDD1 expression was equally activated in s.c. adipose and epidermis (FigD and Supplementary Fig S1).

### REDD1 protein level is tightly regulated in epidermis and correlates with mTOR activity and autophagy

**Figure 2:**
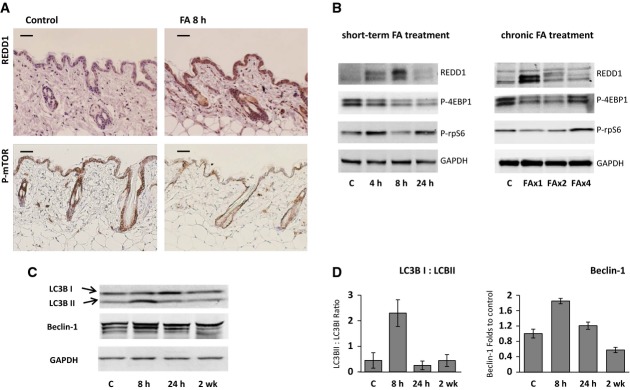
REDD1 protein expression in epidermis is tightly regulated and correlates with inhibition of mTOR and autophagy B6D2 mice were treated topically as in Fig1. Skin was harvested 4–24 h after single FA application or during chronic treatment (8 h after 1^st^, 2^nd^, and 4^th^ applications).A Immunohistochemical staining of mouse skin treated with acetone or FA for 8 h for REDD1 (upper panels) and phosphorylated mTOR-Ser2448 (lower panels). Scale bars are 20 μm.B Western blot analysis of REDD1 protein and phosphorylation of down-stream mTOR target proteins 4E-BP1 and rpS6 in murine epidermis. GAPDH is used as a normalization control.C, D Glucocorticoids induce autophagy in epidermis. Western blot analysis of Beclin-1 and conversion of light chain 3 (LC3) from LC3-I to LC3-II (C). Quantification of LC3-I to LC3-II conversion and Beclin-1 expression (D). The means ± SD were calculated using Western blots from two independent experiments (each lane is whole-cell protein from three pulled individual samples of epidermis).

Both immunostaining and Western blot analysis revealed that REDD1 protein was barely detectable in the skin of adult wild-type mice (Fig2A and B). FA induced REDD1 expression as early as 4–8 h after first application and following subsequent applications during chronic FA treatment, albeit to a lesser extent (Fig2B). The multiband pattern of REDD1 signal on Western blots was reported previously and may reflect REDD1 phosphorylation (Katiyar *et al*, 2009; Li *et al*, 2012; Regazzetti *et al*, 2012). The increase in REDD1 protein in the skin in response to FA was confirmed by immunostaining, which showed that REDD1 was mostly expressed in the cytoplasm of mouse keratinocytes throughout the epidermis (Fig2A).

The observed differences between REDD1 induction dynamics at mRNA and protein levels (especially at the 24-h time point and at the end of chronic FA treatment, compare Fig1C and D, and Fig2B) are likely due its short half-life (Katiyar *et al*, 2009). REDD1 expression is regulated via multiple mechanisms including translational repression by miR-221 which was reported to regulate REDD1 in murine hepatic progenitor cells (Pineau *et al*, 2010). In agreement, pri-miR-221 was significantly increased at the time points when REDD1 protein levels started to decline, for example, 24 h after single FA application, and after the first week of the chronic treatment (Supplementary Fig S5). This inverse correlation suggests that mature miR-221 is possibly involved in control of REDD1 induction by steroids in skin.

Because REDD1 is a known inhibitor of mTOR signaling (Sofer *et al*, 2005; Wang *et al*, 2006; DeYoung *et al*, 2008; Shimizu *et al*, 2011), we tested whether FA inhibits mTOR activity in the skin. In previous studies, mTOR activity has been monitored by phosphorylation of its major substrates, 4E-BP1 (eukaryotic initiation factor 4E binding protein 1) and S6K1 (ribosomal p70/S6 kinase 1) as well as ribosomal protein S6 (rpS6), a substrate of S6K1 (Checkley *et al*, 2011). As basal level of S6K1 phosphorylation was low in skin of B6D2 mice, we used 4E-BP1 and rpS6 as preferred markers. We showed that FA blocked mTOR activity in the skin. Moreover, there was an inverse correlation between phosphorylation of mTOR targets and REDD1 expression in the epidermis. By the time when REDD1 expression reached its peak (8 h after 1^st^ and 2^nd^ FA applications), the repression of mTOR activity by FA was most pronounced (Fig2A and B). We further confirmed this inverse correlation using immunostaining for REDD1 and active, phosphorylated at Ser2448 mTOR (Fig2A).

Previous studies showed that mTOR inhibition by glucocorticoids in other tissues causes autophagy (Molitoris *et al*, 2011; Shimizu *et al*, 2011). In accordance, we observed in FA-treated skin the conversion of light chain 3 (LC3) from its free form (LC3-I) to a membrane-bound state (LC3-II), a key step that initiates autophagy in mammalian cells (Klionsky *et al*, 2012). LC3-I to LC3-II conversion reached its maximum 8 h after FA treatment and paralleled with the increase of another autophagy marker Beclin-1, highest REDD1 expression and strongest repression of mTOR activity (Fig2C and D).

### REDD1 KO mice are resistant to the depletion of stem cells and skin atrophy by glucocorticoids

**Figure 3:**
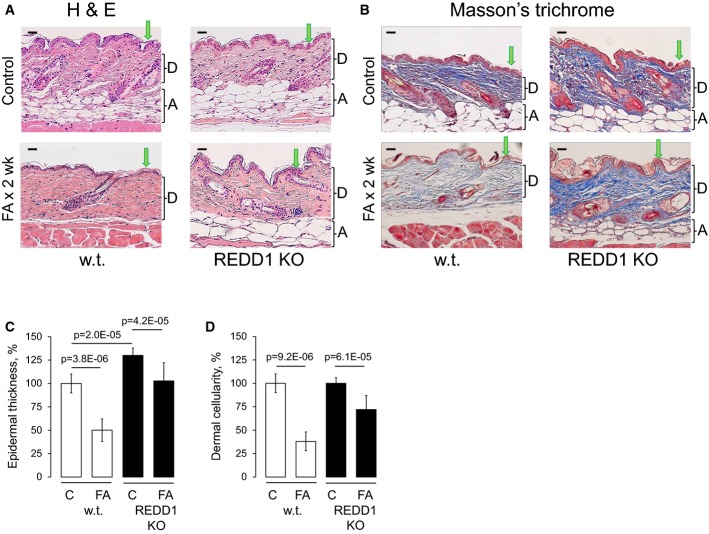
REDD1 KO mice are resistant to glucocorticoid-induced skin atrophy REDD1 KO and isogenic wild-type B6x129 mice were treated with acetone (vehicle control) or FA (2 μg/animal) every 72 h for 2 weeks.A, B H&E staining (A) and Masson's trichrome staining: Dermis/collagen fibers are blue, muscle is red, nuclei are dark red, and cytoplasm is red/pink (B). Arrows point to epidermis and brackets indicate subcutaneous adipose (A) and dermis (D). Scale bars are 20 μm.C, D Morphometric analysis of epidermal thickness and dermal cellularity as described in Materials and Methods. Changes in epidermal thickness (C) are presented as % to wild-type control epidermis. Changes in dermal cellularity (D) are presented as % to corresponding control skin. The means ± SD were calculated for three individual skin samples in one representative experiment (30 measurements/condition) out of two experiments. Statistical analysis for differences between treatment and control and between control wild-type and REDD1 KO epidermal thickness was done by the unpaired two-tailed *t*-test.

To assess the causative role of REDD1 in therapeutic and side effects of glucocorticoids in the skin, we compared REDD1 KO mice (Brafman *et al*, 2004) with isogenic controls (B6x129). REDD1 KO mice displayed mild epidermal hyperplasia and slightly increased keratinocyte proliferation (Fig3C and data not shown). There were no significant changes in early and medium/late differentiation markers including keratins K5, K10, loricrin, and involucrin (Supplementary Fig S2).

In contrast, the lack of REDD1 strongly attenuated the effects of glucocorticoids in the skin. REDD1 KO animals showed considerable resistance to FA-induced epidermal atrophy compared to wild-type animals. In wild-type mice, chronic FA treatment reduced epidermal thickness by 50%, compared to < 20% in REDD1 KO mice (Fig3A and C). Similarly, s.c, adipose tissue was significantly protected from the atrophy upon REDD1 knockout (Fig3A and B). Glucocorticoids induce severe thinning of collagen and elastin fibrous network and decrease cellularity of the dermis (Woodbury & Kligman, 1992; Schoepe *et al*, 2006). Using Masson's trichrome staining, we showed that REDD1 KO minimized FA effect on dermal fibers and caused significant protection of the dermal cells (Fig3B and D, and higher magnification images in Supplementary Fig S6).

**Figure 4:**
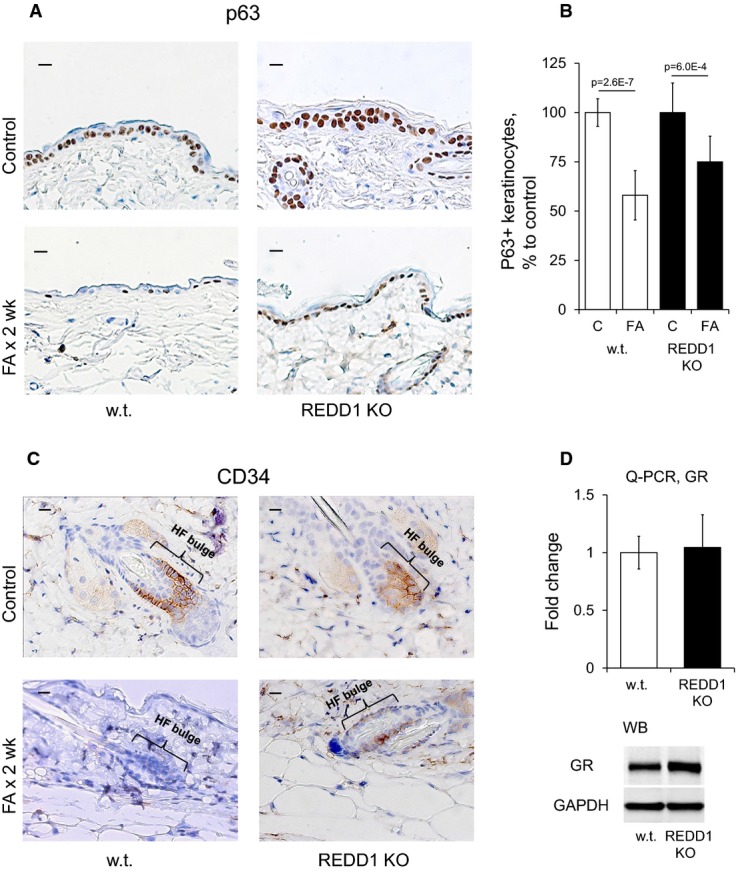
Protective effect of REDD1 KO on p63^+^ progenitors and CD34^+^ follicular epithelial stem cells REDD1 KO and wild-type animals were treated as in Fig3.A–C Expression of p63 (A) and CD34 (C). Scale bars are 10 μm. Analysis of p63 staining (B). The number of p63^+^ basal keratinocytes/total number of basal keratinocytes is presented as % to the corresponding control epidermis.D Similar GR expression in epidermis of wild-type and REDD1 KO mice determined by Q-PCR and Western blotting. Rpl27 and GAPDH used as a normalization controls, respectively.
Data information: The means ± SD were calculated for three individual skin samples in one representative experiment (30 measurements/condition) out of two experiments. Q-PCR results are presented as the means ± SD for three individual RNA samples/condition. Statistical analysis for differences between treatment and control was done by the unpaired two-tailed *t*-test.

Our previous studies showed that GR activation has a profound negative effect on epidermal stem cells (SCs) in the bulge of hair follicles (Chebotaev *et al*, 2007b,c). In agreement, chronic FA treatment of wild-type mice completely eliminated the CD34^+^ follicular SCs (only 5% of hair follicles were CD34 positive) and diminished by ∼40% the numbers of p63-positive progenitors in the basal layer of epidermis (Fig4A and C). Importantly, in REDD1 KO animals treated with FA, CD34^+^ SCs and p63^+^ keratinocytes were largely preserved: 40% of hair follicles remained CD34 positive, and the number of p63^+^ basal keratinocytes decreased only by 20% (Fig4A and C).

### REDD1 KO mice retain sensitivity to the anti-inflammatory effect of glucocorticoids

**Figure 5:**
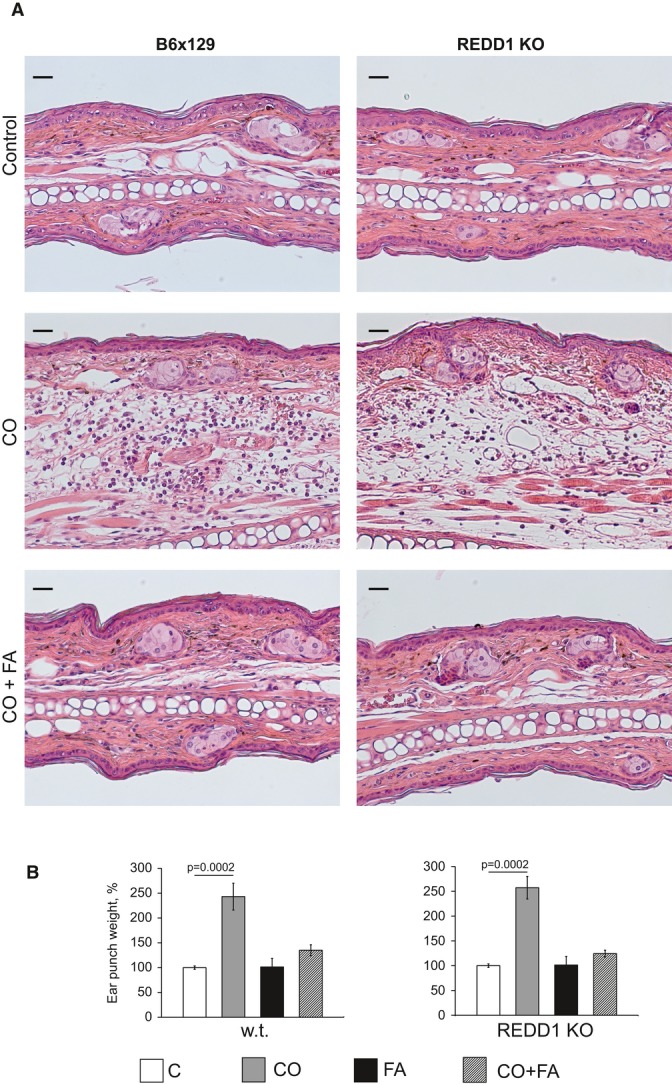
Similar sensitivity of REDD1 KO and wild-type animals to the anti-inflammatory effect of glucocorticoids Ear edema was induced by croton oil (CO) as in Materials and Methods. FA was applied 1 h before CO, and four-millimeter ear punch was weighed 9 h after CO application to assess swelling. In the additional experiment, ears were harvested 9 h after CO application and used for histological analysis.A H&E staining. Scale bars are 20 μm.B Ear punch weight. Results are presented as % to corresponding (wild-type or REDD1 KO) control ear weight. The means ± SD were calculated for six individual ear punches/condition in one representative experiment (out of three experiments). Statistical analysis for differences between treatment and corresponding control was done by the unpaired two-tailed *t*-test.

To further assess the consequences of REDD1 knockout for glucocorticoid therapy, we measured their responses to FA in a model where inflammation/edema is induced by topical irritant croton oil (CO) in the mouse ear. This assay is typically employed to test anti-inflammatory effects of GR ligands (Schäcke *et al*, 2004; Park *et al*, 2006; Schoepe *et al*, 2010). REDD1 KO animals and isogenic controls were equally responsive to inflammation (Fig5). Remarkably, FA relieved this inflammatory response with equal potency in wild-type and REDD1 KO mice as assessed by changes in ear morphology and the weight of ear punch (Fig5).

### REDD1 knockdown reduces hypoplastic effect of glucocorticoids in organotypic raft cultures

**Figure 6:**
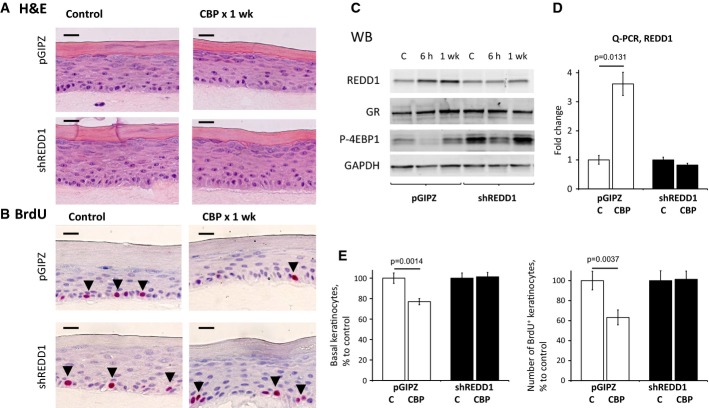
Down-regulation of REDD1 protects organotypic raft cultures from the hypoplastic effect of glucocorticoids Organotypic raft cultures (ORC) made from NHEK infected with REDD1 shRNA and control pGIPZ lentiviruses were treated with glucocorticoid CBP (5 μM) or vehicle control (0.05% DMSO) for 7 days.A, B H&E and BrdU staining of raft cultures (BrdU^+^ cells are indicated by arrowheads). Scale bars are 10 μm.C Western blot analysis of REDD1, GR, and mTOR substrate 4E-BP1 phosphorylation. GAPDH was used as a loading control.D Q-PCR analysis of REDD1 expression in rafts.E Analysis of CBP effect on basal keratinocyte number (left) and keratinocyte proliferation (number of BrdU^+^ basal keratinocytes/total number of basal keratinocytes, right) is presented as % to corresponding control rafts.
Data information: The means ± SD for BrdU^+^ cells and basal keratinocytes in (E) were calculated for two individual rafts in one representative experiment (20 measurements/condition) out of two experiments. Q-PCR results in (D) are the means ± SD calculated for two individual RNA samples/condition. Statistical analysis for differences between treatment and control was done by the unpaired two-tailed *t*-test.

To evaluate possible contribution of REDD1 to glucocorticoids' action in human skin, we used 3-dimensional organotypic raft cultures (ORC) made of primary human epidermal keratinocytes (NHEK, Getsios *et al*, 2009) as described previously (Schoepe *et al*, 2010). We prepared ORCs from NHEK infected with control pGIPZ- or shREDD1-expressing lentiviruses (Fig6A). Both short-term (6 h) and chronic (7 days) treatment with CBP induced REDD1 mRNA and protein in control ORCs; this induction was completely abolished in shREDD1 ORCs (Fig5C and D). In agreement, mTOR activity was increased in shREDD1-expressing rafts compared to pGIPZ controls, with or without CBP treatment, as was evidenced by increased 4E-BP1 phosphorylation (Fig6C).

In pGIPZ-infected ORCs, the number and especially proliferation (BrdU incorporation) of basal keratinocytes were decreased by 25–40% after 7-day treatment with CBP (Fig6A, B and E). In contrast, shREDD1-expressing ORCs were completely protected from the hypoplastic/anti-proliferative effect of CBP (Fig6A, B, and E).

### REDD1 plays an important role in GR signaling: asymmetrical effect on GR TA and TR

The reduced sensitivity of REDD1 KO mice and shREDD1 ORCs to steroid-induced atrophy suggested that lack of REDD1 could affect either GR expression or GR function. Q-PCR and Western blotting showed that GR expression was not significantly changed/slightly increased either in the epidermis of REDD1 KO mice (Fig4D) or in ORCs after shREDD1 knockdown (Fig6C).

To investigate the global role of REDD1 in GR signaling, we performed genome-wide expression profiling of the epidermis of REDD1 and wild-type mice treated with vehicle (control) or FA using Illumina mouse whole-genome gene array (GEO Submission GSE59151). Gene expression was measured 24 h post-FA application, when both TA and TR are usually fully developed (Wu *et al*, 2004). Differential expression was identified based on the adjusted *P*-value threshold of 0.05.

**Figure 7:**
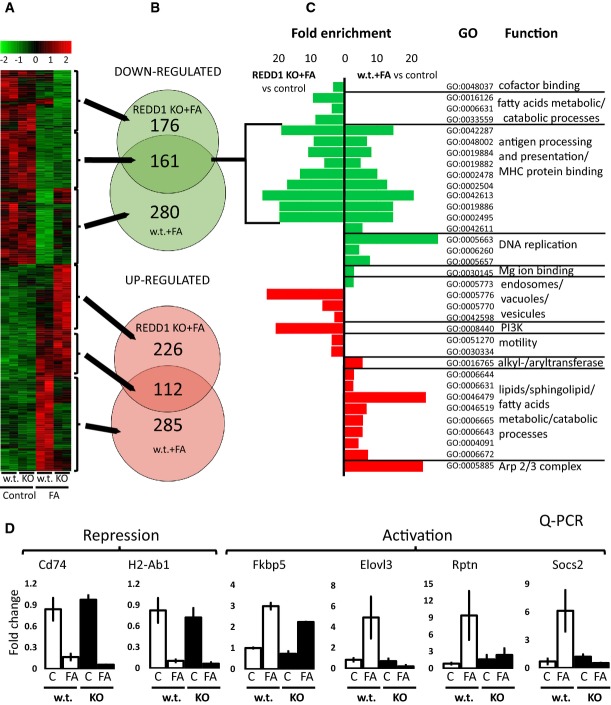
Global effect of REDD1 KO on the expression of glucocorticoid-responsive genes in murine epidermis REDD1 KO and isogenic B6x129 mice were treated topically with FA (2 µg) or vehicle (acetone) for 24 h, and RNA was extracted from the epidermis and used for microarray analysis.A A heatmap of gene expression as analyzed by the Mouse Whole-Genome Gene Expression BeadChips (Illumina). Columns show normalized gene expression for individual animals (red: increased, green: decreased expression).B Venn diagrams show overlap of differentially expressed genes between REDD1 KO and wild-type mice (adjusted *P* < 0.05). Arrows connect sections of the heatmap with corresponding Venn diagrams.C Most enriched gene ontology (GO) categories for biological processes and molecular functions (fold-change enrichment ≥ 3, *P* < 0.01), associated with transrepression and transactivation of the glucocorticoid-responsive genes in REDD1 KO and wild-type mice.D Array validation by Q-PCR for genes from the different GO categories. Note: lack of overlap in GO categories between KO and wild-type mice associated with up-regulated genes and significant overlap in the function of down-regulated genes.
Data information: We used for array analysis two individual RNA samples/condition. Statistical analysis of DNA arrays is described in Materials and Methods. Statistical analysis of array validation is shown in Supplementary Fig S3.

REDD1 KO had only minor effect on baseline gene expression with only three genes were up-regulated and 23 were down-regulated in REDD1 KO compared to wild-type controls (Supplementary Table S1, and heatmaps Fig7A and Supplementary Fig S4).

In contrast, REDD1 appeared central for gene regulation by glucocorticoids. In REDD1 KO animals, ∼20% fewer genes responded to FA than in wild-type mice: (338 versus 397 up-regulated and 337 versus 441 down-regulated, Fig7B and Supplementary Table S2). More importantly, the lack of REDD1 specifically altered TA branch of GR signaling. Only 30% FA-induced genes were also activated in REDD1 KO mice. In contrast, there was a 50% overlap between genes inhibited by FA in REDD1 KO and wild-type animals (Fig7B). The detailed analysis of differentially regulated individual genes in two genotypes is presented in Supplementary Table S2.

To quantify the relationship between TA and TR and REDD1 gene status, we assessed Pearson correlation between the fold changes of the differentially expressed (397 activated and 441 down-regulated) genes in wild-type and in REDD1 KO epidermis. We found only a weak positive correlation between FA-induced genes (*r* = 0.16, *P* = 0.003), and a robust positive correlation between FA-inhibited genes (*r* = 0.72, *P* = 10e-10), indicating much stronger involvement of REDD1 in GR TA, but not TR. This conclusion is also illustrated by the hierarchical clustering of microarray data on the heatmap with high level of similarity between genes repressed by FA in wild-type and REDD1 KO animals (Fig7A, Supplementary Fig S4).

Next, we performed Gene Ontology (GO) enrichment analysis of the differentially expressed genes for each comparison (Supplementary Table S3). The most enriched GO terms associated with the up-regulated genes in wild-type epidermis included metabolic processes (lipids, sphingolipids, and fatty acids), catabolism (lipids, sphingolipids, proteins), and proteolysis. These metabolic and catabolic gene categories were absent among genes up-regulated in REDD1 KO epidermis (Fig7C). Instead, in REDD1 KO keratinocytes, FA up-regulated genes related to autophagy, endosomes, and PI3K signaling. Remarkably, the GO analysis of gene repression showed 70% overlap between REDD1 KO and wild-type animal response, with predominant categories related to the anti-inflammatory effect of glucocorticoids (antigen processing/presentation, and MHC/major histocompatibility complex protein binding) (Fig7C). In addition, FA inhibited genes related to DNA replication, reflective of the anti-proliferative effect of glucocorticoids in wild-type but not in REDD1 KO skin.

Array validation was performed by Q-PCR for six genes from different GO categories, inhibited (Cd74, H2-Ab1) or up-regulated (Fkbp5, Elovl3, Rptn, Socs2) by FA in wild-type epidermis (Fig7D). Remarkably, Cd74 and H2-Ab1 associated with class II MHC and important for antigen presentation and immune response (Pan *et al*, 2001; Beswick & Reyes, 2009) were similarly down-regulated in wild-type and in REDD1 KO epidermis (Fig7D). The examples of genes differentially activated in wild-type and REDD1 KO epidermis include Elovl3 involved in lipid metabolism (Guillou *et al*, 2010), Rptn, a multifunctional matrix protein (De Guzman Strong *et al*, 2010), Socs2, which regulates cell signaling and protein degradation (Larsen & Röpke, 2002), and Fkbp5, a molecular chaperone involved in steroid receptor activation, and also an inhibitor of mTOR and Akt (Li *et al*, 2011; Vandevyver *et al*, 2012) (Fig7D). Q-PCR data for all these genes were in perfect agreement with the DNA array analyses (Pierson correlation > 0.9, Supplementary Fig S3).

Overall, the analysis of gene expression at single gene and GO category levels, for the first time, revealed a feed-forward loop whereby GR target REDD1 is necessary for its TA action.

### REDD1 does not alter GR phosphorylation and nuclear localization

**Figure 8:**
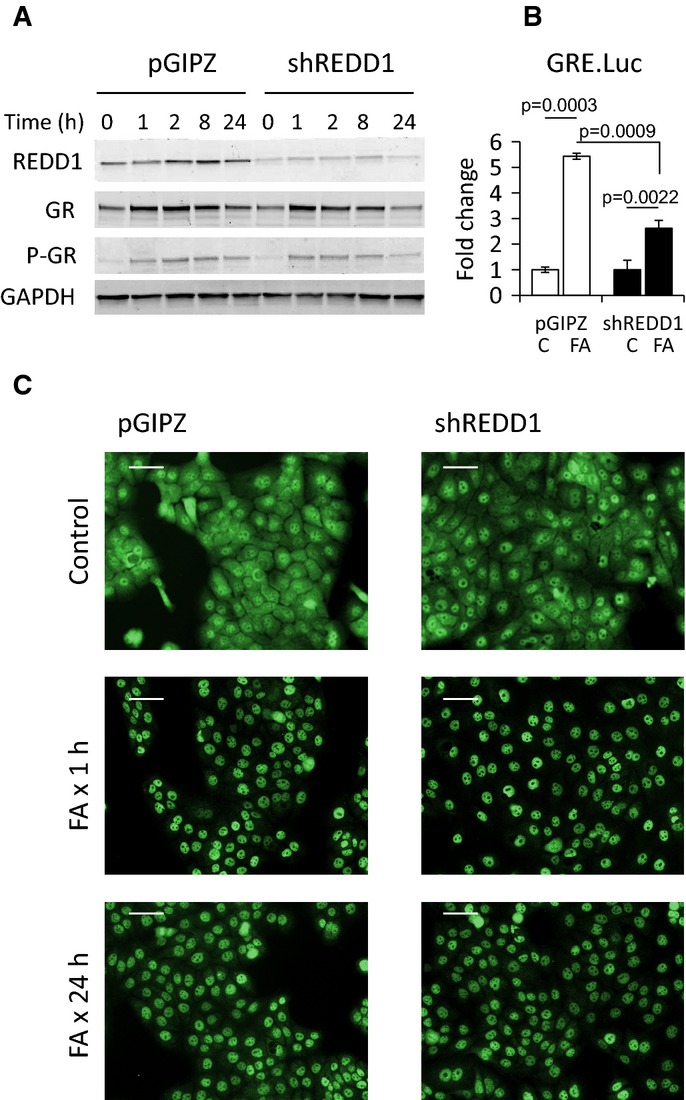
REDD1 inhibition did not affect GR phosphorylation and nuclear translocation in HaCaT human keratinocytes HaCaT human keratinocytes were infected with shREDD1 or pGIPZ (control) lentiviruses and treated with glucocorticoid FA (10^−6^ M) for the indicated time.Western blot analysis of REDD1, GR, and phosphorylated GR-Ser211. GAPDH used as a loading control.Reduced induction of Luciferase reporter in shREDD1-HaCaT cells. shREDD1- and pGIPZ-HaCaT cells were infected with GRE.Luc lentivirus and treated with FA (10^−6^ M) for 24 h. The Luciferase induction is presented as a fold change to corresponding vehicle-treated control. The means ± SD were calculated for three individual wells/group in one representative experiment (out of three experiments). Statistical analysis for differences between groups was done by ANOVA.Immunofluorescence analysis of GR nuclear translocation and retention in shREDD1- and pGIPZ-HaCaT cells treated with FA (10^−6^ M). Scale bars are 10 μm.

The major steps required for GR-dependent TA are phosphorylation and nuclear translocation. ORC are notoriously difficult for biochemical analyses using multiple time points. Thus, we used immortalized human keratinocytes (HaCaT), stably infected with control pGIPZ or shREDD1 lentiviruses, in which shREDD1 prevented REDD1 activation by glucocorticoid FA (Fig8A).

In agreement with the impaired activation of GR target genes in REDD1 KO animals, GR activation was strongly decreased by REDD1 knockdown in HaCaT keratinocytes as assessed by Luciferase assay with GRE.Luciferase reporter (Fig8B). Upon ligand binding, GR is typically degraded; however, in some cells, this degradation is minimal and may be preceded by temporary GR accumulation (Yemelyanov *et al*, 2008). We observed this temporary GR accumulation in HaCaT cells (Fig8A). Despite the difference in GR activity in Luciferase assay, basal GR levels, the dynamics of GR protein changes, GR phosphorylation, and nuclear translocation in response to FA were identical in shREDD1- and pGIPZ-HaCaT keratinocytes (Fig8A and C).

### Similar mechanisms control skin and muscle atrophy

Glucocorticoids can induce catabolic/atrophic changes in multiple tissues including muscle, subcutaneous fat, and bone (Wang *et al*, 2006; Shimizu *et al*, 2011). We used ProfileChaser software to compare transcriptional responses to glucocorticoids in epidermis and other tissues, using our array data and DNA arrays deposited to NCBI (http://profilechaser.stanford.edu, Engreitz *et al*, 2010; Dudley *et al*, 2011). The comparison revealed remarkably high degree of similarity between gene expression patterns in steroid responses of the epidermis and muscle and, unexpectedly, steroid-independent changes in Duchene muscular dystrophy (similarity scores 0.66 and 0.64 accordingly, Table1).

**Table 1:** Similar molecular signature of GR in epidermis, s.c. adipose and in muscle

Skin compartment	GEO	Study title	Organism	Similarity score	*Q*-value
Epidermis	GDS2688	Skeletal muscle response to chronic glucocorticoids	*Rattus norvegicus*	0.657	0.003
Epidermis	GDS3027	Duchenne muscular dystrophy	*Homo sapiens*	0.64	0.004

Gene expression in epidermis of wild-type mice treated with FA was analyzed by Illumina oligo arrays (as in Fig6). The gene expression changes in epidermis were compared to the gene expression profiles from publicly available experiments obtained from NCBI Gene Expression Omnibus (GEO) by ProfileChaser software (Stanford University, http://profilechaser.stanford.edu/). The Similarity Score is a weighted Pearson correlation coefficient between arrays. *Q*-value = FDR, false discovery rate.

## Discussion

This work represents comprehensive investigation of the mechanisms of adverse and therapeutic glucocorticoid effects in skin, which combines focused and global, genome-wide methods of analysis.

First, we shed new light on the dynamics of molecular responses to topical glucocorticoids in the skin. We have identified REDD1 as an early glucocorticoid-responsive gene in mammalian skin. REDD1 activation in mice occurs 4–8 h after the treatment when it inhibits mTOR and promotes autophagy in the epidermis. Later, at the end of 2-week glucocorticoid treatment, both REDD1 and mTOR activity return to basal levels (Fig2B). This is the time point when mice develop resistance to topical glucocorticoids (tachyphylaxis) (Chebotaev *et al*, 2007c). In contrast to mice, tachyphylaxis does not occur in patients (Taheri *et al*, 2013); this could be possibly caused by persistence of REDD1 up-regulation.

This is the first study in which REDD1 KO mice were used to determine the contribution of REDD1 to skin maintenance and response to glucocorticoids. In general, REDD1 role in skin and keratinocytes has not been studied; a single report suggests increased keratinocyte differentiation *in vitro* due to REDD1 (Ellisen *et al*, 2002). In our study, REDD1 had minimal effect on the gene expression in adult epidermis, and this is corroborated by the fact that adult REDD1 KO animals have no overt skin phenotype, likely due to low basal REDD1 levels.

At the same time, REDD1 induction by glucocorticoids was critically important for skin atrophy. In REDD1 KO mice, all skin compartments were protected from the atrophogenic effect of steroids. Likewise, ORCs of human epidermis, which are currently the most advanced model of human skin (Getsios *et al*, 2009; Schoepe *et al*, 2010), were also protected from hypoplasia by REDD1 knockdown. In contrast to its central role in adverse cutaneous effects of steroids, REDD1 is dispensable for the anti-inflammatory effect of glucocorticoids as FA alleviated inflammation and edema due to croton oil in both REDD1 KO and control mice.

Interestingly, REDD1 knockout safeguarded at least two populations of stem cells: CD34^+^ follicular epithelial stem cells and p63^+^ keratinocyte progenitors from the detrimental effects of glucocorticoids. These data along with recent observations that REDD1 is decreased during reprogramming of somatic cells to induced pluripotent stem cells (Corominas-Faja *et al*, 2013) suggest an important role for REDD1 in stem cells maintenance. CD34 is also a marker of white adipose stem cells (Park *et al*, 2008), and thus, protection of CD34^+^ adipocytes could explain the observed protection of s.c. fat in REDD1 KOs.

Recent report documents the inhibitory effect of mTOR on GR function in the muscle (Shimizu *et al*, 2011); however, the effect of REDD1 and other mTOR inhibitors on GR signaling has not been well studied. Our bioinformatics analysis of transcriptional response to glucocorticoids in the skin identified a novel intriguing role of REDD1 in control of the repertoire of glucocorticoid-regulated genes and the integral functional response to glucocorticoids. Furthermore, the lack of REDD1 predominantly affected gene activation (TA) and not inhibition (TR) by glucocorticoids.

While TA involves binding of GR homodimers to palindromic GREs in the promoters and enhancers of the glucocorticoid-inducible genes, TR is largely independent of GR dimerization and in many cases stems from the direct interaction (tethering) between GR monomer and another transcription factors including pro-inflammatory NF-kB and AP-1. Another TR mechanism is binding of GR monomers to ‘negative GREs’ (Nixon *et al*, 2013; Ratman *et al*, 2013). The TA versus TR outcome also depends on the recruitment of steroid hormone receptor coactivators (such as SRC-1, SRC2) and corepressors (such as NCoR, SMRT) (Ratman *et al*, 2013). As GR phosphorylation and nuclear import/retention were similar in control and shREDD1-HaCaT keratinocytes, it is conceivable that REDD1 modifies conditions for GR dimerization and DNA binding, or impinges on the specter of coregulators recruited by GR.

TR by GR is critical for its anti-inflammatory function, even though the induction of select GR-dependent genes is also needed (Schäcke *et al*, 2006, 2009; Chebotaev *et al*, 2007a; Clark *et al*, 2008; Ratman *et al*, 2013). In contrast, many side effects of GR are caused by its TA action (Schäcke *et al*, 2006, 2009; Ratman *et al*, 2013). Thus, selective GR activators (SEGRA) that shift GR activity toward TR are expected to have a better therapeutic profile than classical glucocorticoids. Indeed, several SEGRA including ZK245186/mapracorat preserve the anti-inflammatory effect of glucocorticoids but do not induce skin atrophy (Schäcke *et al*, 2009). We demonstrate that REDD1 deletion ‘dissociates’ TA and TR functions of glucocorticoids.

Chronic glucocorticoid therapy induces atrophy in many organs besides skin (Wang *et al*, 2006; Shimizu *et al*, 2011; Henneicke *et al*, 2014). Meta-analysis of ours and published gene arrays revealed strong similarity between changes in transcriptome in glucocorticoid-treated epidermis (our results) and muscle undergoing steroid-dependent and steroid-independent (Duchene atrophy) waste. Thus, molecular mechanisms underlying the atrophy in epidermis and muscle are likely similar and involve REDD1. We also speculate that similar REDD1-dependent mechanisms underlie the steroid atrophy of s.c. adipose as REDD1 was highly induced in s.c. adipocytes, and s.c. fat was protected in REDD1 KO mice from glucocorticoids.

In conclusion, we discovered that REDD1 acts as GR modulator and atrophogen in the skin. Further, our results suggest the clinical relevance of REDD1 as molecular target for safer combination GR-targeted therapies in the skin. We expect that blocking REDD1 by pharmacological inhibitors or RNAi could reduce and possibly alleviate skin atrophy in response to steroids; this is a new strategy for safer glucocorticoid treatments of chronic inflammatory diseases in the skin and other tissues.

## Materials and Methods

### Chemicals

Fluocinolone acetonide (FA), croton oil (CO), and all other chemicals unless stated otherwise were purchased from Sigma-Aldrich Corp. (St. Louis, MO, USA). Clobetasol propionate (CBP) was purchased at the pharmacy as 0.05% cream.

### Animals and treatments

B6D2 (F1 C57Bl ×DBA) mice used previously to study steroid skin atrophy (Chebotaev *et al*, 2007c) were obtained from Jackson Laboratory (Bar Harbor, ME, USA). REDD1 KO mice in F1 C57BL/6 × 129SvEv genetic background (B6x129) were generated by Lexicon Genetics Inc. for Quark Pharmaceuticals Inc. REDD1 exon 2 was replaced with LacZ/neo expression cassette (Brafman *et al*, 2004). Wild-type B6x129 isogenic control females were obtained from Taconic (Germantown, NY, USA).

Seven-week-old females in the telogen stage of the hair cycle were shaved and treated 3 days later. Glucocorticoid FA was applied topically (2 μg/animal) in 200 μl acetone to the back skin once or up to four times every third day as described (Chebotaev *et al*, 2007c). Control animals were treated with acetone only. Skin was harvested 4–24 h after FA application as indicated in Figure legends. Animals were injected i.p. with bromodeoxyuridine (BrdU, Sigma-Aldrich, 50 μg/g of animal weight) 1 h before skin was harvested. Epidermis and s.c. fat were isolated from the murine dorsal skin mechanically by scraping (Chebotaev *et al*, 2007c).

In our work with animals, we adhered to ACUC protocols approved by the Northwestern University Animal Care and Use Committee. The protocols specify experimental procedures (topical treatment with glucocorticoids, ear edema test, BrdU injections), mouse strains (C57Blx129; C57BlxDBA, REDD1 KO), animal sex (males and females), age (from 5 weeks to 1 year), number of animals allowed to use, housing details (five animals/cage, regular chow diet, food and water *ad libitum*, 12 h on/12 h off light exposure), and animal euthanasia. All animals were maintained at the Northwestern University barrier animal facility.

#### Ear edema test

To evaluate the anti-inflammatory effect of glucocorticoid FA, we used ear edema test (Schäcke *et al*, 2004; Park *et al*, 2006; Schoepe *et al*, 2010). Seven-week-old female animals were pretreated with FA (2 μg in 20 μl of acetone) or vehicle (20 μl of acetone) applied to the back of the ear lobe 1 h before application of nonspecific contact irritant croton oil (CO, 10% solution in 20 μl of acetone). Mice were sacrificed and ears were harvested 9 h after CO application, the time point at which we observed maximum ear edema in B6x129 animals (data not shown). Four-millimeter ear punch biopsies were immediately weighted to assess ear edema. In all experiments, we used 3–4 animals/group; experiments were repeated 2–3 times.

### Treatment of human volunteers

Glucocorticoid CBP was applied topically as a 0.05% cream to the skin of right arm of healthy human volunteers (age 32–65) once or every 24 h for 2 weeks. Untreated left arm skin was used as control. Four-millimeter-full-thickness punch skin biopsies were taken 24 h after the last CBP application as indicated in Figure legends. Whole human skin samples were used for the molecular biological studies.

### Keratinocyte cell lines and 3-D organotypic raft cultures

Three-dimensional (3-D) organotypic raft cultures (ORC) of human epidermis were made as described in Getsios *et al* (2009). Briefly, neonatal human epidermal keratinocytes (NHEK) were infected with shRNA- and pGIPZ-expressing lentiviruses followed, 48 h later, by selection for puromycin (2 μg/ml) resistance. Selected keratinocyte cultures were reseeded onto collagen gels with embedded J2-3T3 fibroblasts and cultured at the air–liquid interface as previously described for 3 days to allow the initial phase of epidermis formation. The standard ORC medium contains high 5 × 10^−7^ M hydrocortisone. In our experiments, ORC were cultured in the medium with 10^−8^ hydrocortisone during first 3 days and then were treated with glucocorticoid CBP (5 × 10^−6^ M) or vehicle control (0.05% DMSO) for 7 days. Rafts were treated with BrdU (10^−7^ M) 1 h before harvesting and fixed in formalin. The epithelial sheets were peeled off the collagen lattice, snap-frozen, protein and RNA extracted, and processed for Western blot analysis and Q-PCR.

HaCaT human keratinocyte cell line is an *in vitro* spontaneously transformed keratinocytes from histologically normal skin. Line was established by Dr. Fusenig (Boukamp *et al*, 1988). We have obtained HaCaT cells from Dr. M Denning (Loyola University, Chicago, IL) who received them directly from Dr. Fusenig. It is known that HaCaTs express basal keratin K14 and can express keratins K1/K10 when induced to differentiate by high Ca^2+^ (Boukamp *et al*, 1988). We confirmed that our subline of HaCaT cells express keratinocyte differentiation markers using Q-PCR (data not shown). Cells were cultured in Dulbecco's modified Eagle's medium (DMEM, Cellgro, Manassas, VA, USA) containing 10% FBS (Cellgro) and antibiotics.

### Lentiviruses

#### shREDD1-lentiviral construct

To knockdown REDD1 expression in keratinocytes, we used lentiviral construct expressing shREDD1 targeting REDD1 3′ UTR sequences homologous in human and mouse (clone V2LHS_176476, Thermo Scientific GIPZ Lentiviral shRNA Library). Empty pGIPZ vector (Open Biosystems, GE Healthcare Bio-Sciences, Pittsburgh, PA, USA) was used as a control.

#### GRE.Luciferase Reporter

GRE.Luc reporter lentiviral construct encoding Firefly Luciferase under transcriptional regulation of glucocorticoid-responsive elements and a control lentiviral construct with Firefly Luciferase under minimal CMV promoter (used as control) were obtained from DNA/RNA delivery Core, SDRC Northwestern University. Lentiviral stocks, packaging, and transduction procedures were performed as described in Zufferey *et al* (1998) and Yemelyanov *et al* (2007).

### Luciferase assay

HaCat cells expressing Firefly Luciferase under minimal CMV promoter or promoter containing GRE were plated in 12-well plates (three wells/experimental group), grown to ∼60% and treated with FA or vehicle (0.01% DMSO) for 24 h. Luciferase activity was measured using commercial Luciferase Assay (Promega Corp., Madison, WI, USA) and Luminometer TD 20/20 (Turner Designs, Sunnyvale, CA, USA). Luciferase activity of GRE.Luc construct was normalized to Luciferase activity from minimal CMV promoter under the same experimental condition.

### Histological analysis and immunostaining

Sections of formalin-fixed, paraffin-embedded skin and ORCs were stained with hematoxylin and eosin (H&E), Masson's trichrome to evaluate the effect on dermis and collagen fibers (Sheehan & Hrapchack, 1980), and with antibodies against BrdU (BD Biosciences, San Jose, CA, USA), keratins 1, 5, and 10, loricrin (Covance, Princeton, NJ), phospho-mTOR^Ser2448^ (Cell Signaling Technology, Inc., Danvers, MA, USA), REDD1 (Proteintech Group, Inc, Chicago, IL), p63 (eBioscience, San Diego, CA, USA), and CD34 (Abcam, Cambridge, MA, USA).

GR nuclear translocation was determined by immunofluorescence in HaCaT cells. Cells infected with pGIPZ and shREDD1 lentiviruses were selected with puromycin, seeded on coverslips. After the treatments, cells were fixed with 2% formaldehyde and permeabilized with acetone:methanol (1:1 v/v). After blocking, cells were incubated with primary rabbit anti-GR antibody (H-300, Santa Cruz Biotechnology, Santa Cruz, CA, USA) followed by secondary anti-rabbit FITC-conjugated antibody (Jackson Immuno Research). Cell nuclei were counterstained with DAPI (Invitrogen, Life Technologies, Grand Island, NY, USA). Cell and tissue images were taken with AxioCaM HRC camera linked with Zeiss Axioplan2 microscope.

### Morphometric analysis

Quantification of the epidermal width and number of basal keratinocytes (as the readouts for skin atrophy and hypoplasia of raft epidermis) was performed in dorsal skin and ORC sections stained with H&E. The number of dermal cells was determined on sections stained with Masson's trichrome in the upper (papillary) dermis that is distinguishable from the lower (reticular) dermis (Driskell *et al*, 2013). At least 10 individual fields per slide with at least three samples in each experimental group were counted using Axioplan2 microscope software (Carl Zeiss). All measurements are presented as % to corresponding control.

The numbers of BrdU^+^, p63^+^, and total number of basal keratinocytes were evaluated in 10 fields of view in each skin/ORC sample under the microscope. Number of BrdU^+^ and p63^+^ cells is presented as percent of total number of basal keratinocytes. The number of CD34^+^ hair follicles among 10 randomly selected hair follicles per skin sample was evaluated under the microscope.

### Western blot analysis

The whole-cell protein extracts were prepared using RIPA buffer with protease and phosphatase inhibitor cocktails (Thermo Scientific, Thermo Fisher Scientific Inc., Waltham, MA, USA), resolved by SDS–PAGE on 4–20% gels and transferred to Odyssey nitrocellulose membranes (LI-COR Biosciences, Lincoln, NE, USA). Membranes were blocked with Odyssey Blocking Buffer and incubated with primary antibodies overnight at 4°C, followed by IRDye® secondary antibodies (LI-COR Biosciences). LI-COR Odyssey Imager was used for the band visualization. Equal loading and adequate transfer to the membranes were verified by staining with Ponceau S (Sigma-Aldrich) and with anti-GAPDH (Sigma-Aldrich) antibody. We used Abs against: REDD1 (Proteintech Group, Inc., Chicago, IL), LC3B, phospho-rpS6^Ser240/244^, phospho-4E-BP1^Thr37/46^, phospho-GR^Ser211^ (Cell Signaling Technology, Inc.), and GR (H-300 or M-20, Santa Cruz Biotechnology, Inc.).

Abs against REDD1 and Beclin-1 (Fig2B and C) were reported to recognize multiband pattern on Western blots (Katiyar *et al*, 2009; Li *et al*, 2012; Regazzetti *et al*, 2012, and http://www.cellsignal.com/products/primary-antibodies/3738?Ntt=beclin&fromPage=plp), which may reflect phosphorylation status of these proteins.

### RNA isolation and quantitative and semi-quantitative RT–PCR

Total RNA from murine epidermis, whole human skin, and cell cultures was isolated with RiboPure kit (Ambion, Life Technologies, Grand Island, NY, USA). Total RNA from murine s.c. adipose was isolated with RNeasy Lipid Tissue Kit (Qiagen, Valencia, CA, USA). The RNA samples were treated with TURBO™ DNase (Ambion). The gene expression was assessed using semi-quantitative two-step RT–PCR and quantitative Q-PCR. Reverse transcription was performed using 1 μg RNA, random hexamers, and M-MLV reverse transcriptase (Invitrogen, Life Technologies), according to manufacturer instructions. The gene-specific primers were designed with NCBI Primer-BLAST (Supplementary Table S4). Q-PCR with SYBR Green detection was performed on the Applied Biosystems® 7000 Real-Time PCR instrument (Life Technologies). Each sample was tested in triplicate, and results were normalized to the expression of the housekeeping Rpl27 gene (de Jonge *et al*, 2007). For semi-quantitative PCRs, Taq DNA polymerase (Promega) was used, and PCR products were separated on 1.5% agarose gel and visualized using automated imaging system (Bio-Rad, Hercules, CA, USA). RPL27 was used as a normalization control.

### Microarray analysis of gene expression

RNA samples were checked for quality and integrity with the Agilent 2100 bioanalyzer and used for microarray analysis. RNA amplification, labeling, and hybridization with the Mouse Whole-Genome Gene Expression BeadChips MouseRef-8 v2.0 (Illumina) were performed at the Genomics Core Facility at the Center for Genetic Medicine at Northwestern University according to Illumina protocols.

Microarray processing was performed using the Limma package (Smyth, 2005), using the neqc function to perform background subtraction using the negative control probes, and quantile normalization using both positive and negative control probes. Differentially expressed probes were identified using the linear model implemented in Limma, applying an adjusted *P*-value threshold of 0.05. Q-PCR and microarray based gene expression values were compared using linear Pearson correlation.

### FA-induced transactivation and transrepression

To quantify the relationship between FA-induced transactivation and transrepression across both phenotypes, we correlated the fold change of the differentially expressed probesets in wild-type, with their corresponding values in REDD1 KO epidermis.

### Gene Ontology and pathway analysis

Gene ontology analysis was performed using the DAVID Bioinformatics resource tool (Huang *et al*, 2009a,b), submitting differentially expressed probes from each experiment, against the probe background of MouseRef 8. DAVID performs a hypergeometric test to identify overrepresented GO terms in a list of differentially expressed probes. Results are presented for categories with greater than or equal to threefold enrichment and *P* ≤ 0,01.

### Gene expression heatmaps

Heatmaps were generated using differentially expressed probesets (adjusted *P* ≤ 0.05) induced by the application of FA, for the REDD1 KO and wild-type isogenic mice. Samples and genes were hierarchically clustered according to the Euclidean distance of the normalized gene expression values, and clusters were merged using complete linkage. Heatmaps were visualized using the Pheatmap package (http://CRAN.R-project.org/package=pheatmap).

### Statistical analysis

Mean and standard deviation values were calculated using Microsoft Excel software. The treatment effects in each experiment were compared by one-way ANOVA or *t*-test. Differences between groups were considered significant at *P* < 0.05.

All experiments were repeated two to three times. In animal experiments, we used three to four animals/experimental group. In all figures, the results of one representative experiment are shown as mean values ± SD.

### Approval of animal and human studies

All animal experiments were performed in compliance with ACUC protocol approved by the Northwestern University Animal Care and Use Committee.

All human studies conformed to the principles set out in the WMA Declaration of Helsinki (http://www.wma.net/en/30publications/10policies/b3/) and the NIH Belmont Report (http://www.hhs.gov/ohrp/humansubjects/guidance/belmont.html). Studies were approved by Northwestern University Institutional Review Board. Written informed consent was received from the participants before the study.

**The paper explained**

**Problem**

Millions of patients worldwide are affected by chronic inflammatory skin diseases, including atopic dermatitis and psoriasis. The glucocorticoids are among the most effective and frequently used anti-inflammatory drugs. Unfortunately, patients chronically treated with topical glucocorticoids develop side effects, including cutaneous atrophy which affects all skin compartments and compromises the barrier function of the skin. Skin atrophy after the long-term use of steroids may be irreversible. The molecular mechanisms of steroid-induced skin atrophy are poorly understood, which prevents the development of novel safer approaches to the glucocorticoid-based therapies.

**Results**

In this study, we identified REDD1 (regulated in development and DNA damage response 1), a stress-inducible inhibitor of mTOR, as a major molecular target of glucocorticoids which mediates skin atrophy.

In REDD1 KO mice, all skin compartments (epidermis, dermis, and subcutaneous fat) and skin stem cells were significantly protected from the atrophogenic effect of steroids. Likewise, 3-D organotypic raft cultures made from shREDD1-infected primary human keratinocytes were also protected from glucocorticoid hypoplastic effects. In strong contrast to its central role in cutaneous adverse effects of steroids, REDD1 appeared to be dispensable for their anti-inflammatory effects.

Glucocorticoid effects are mediated by their receptor GR, a well-characterized transcription factor. We identified a novel intriguing role of REDD1 as an important GR modulator. REDD1 controls both the repertoire of glucocorticoid-regulated genes and the integral functional response to glucocorticoids. The lack of REDD1 strongly affected activation of genes (including genes involved in metabolism/catabolism of lipids and proteins) by glucocorticoids. At the same time, the inhibition of pro-inflammatory genes related to the anti-inflammatory effects of glucocorticoids remained remarkably similar in wild-type and REDD1 KO mice.

**Impact**

Our novel observations that in context of REDD1 KO cells GR changes its function could explain the improved therapeutic index of glucocorticoids (preserved therapeutic potential combined with reduced atrophogenic effects) in REDD1 KO animals. Overall, our studies support the development of innovative safer therapies with topical glucocorticoids using REDD1 inhibitors (such as pharmacological inhibitors or siRNA) to reduce/alleviate glucocorticoid-induced skin atrophy. As glucocorticoids are among the most frequently prescribed drugs for the patients with chronic inflammatory diseases and induce atrophogenic effects in different tissues, our findings have important clinical applications beyond the field of dermatology.

## Supporting Information

Supplementary Table S4

Review Process File
