# Supplementary material for: REDD1 functions at the crossroads between the therapeutic and adverse effects of topical glucocorticoids
Source: EMBO Mol Med. 2014 Dec 11;7(1):42–58. doi: 10.15252/emmm.201404601 (PMC4309667; doi:10.15252/emmm.201404601)
Supplement: Supplementary file 1 [file emmm0007-0042-sd1.pdf]

RT-PCR

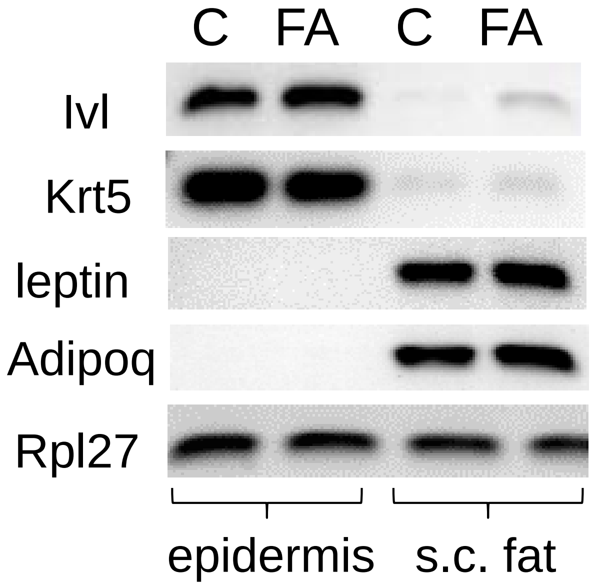

Supplemental Figure 1. Analysis of fat RNA isolation purity. Total RNA from s.c. adipose was isolated, converted to cDNA, and subjected to PCR as described in Materials and Methods. Adipose RNA purity was confirmed by evaluation of expression of epidermal (keratin 5 and involucrin) and adipose (leptin, adiponectin) markers in epidermis and s.c. fat from control B6D2 mice and mice treated with FA for 24 h. Rpl27 was used as a cDNA loading control.
