# Supplementary material for: REDD1 functions at the crossroads between the therapeutic and adverse effects of topical glucocorticoids
Source: EMBO Mol Med. 2014 Dec 11;7(1):42–58. doi: 10.15252/emmm.201404601 (PMC4309667; doi:10.15252/emmm.201404601)
Supplement: Supplementary file 2 [file emmm0007-0042-sd2.pdf]

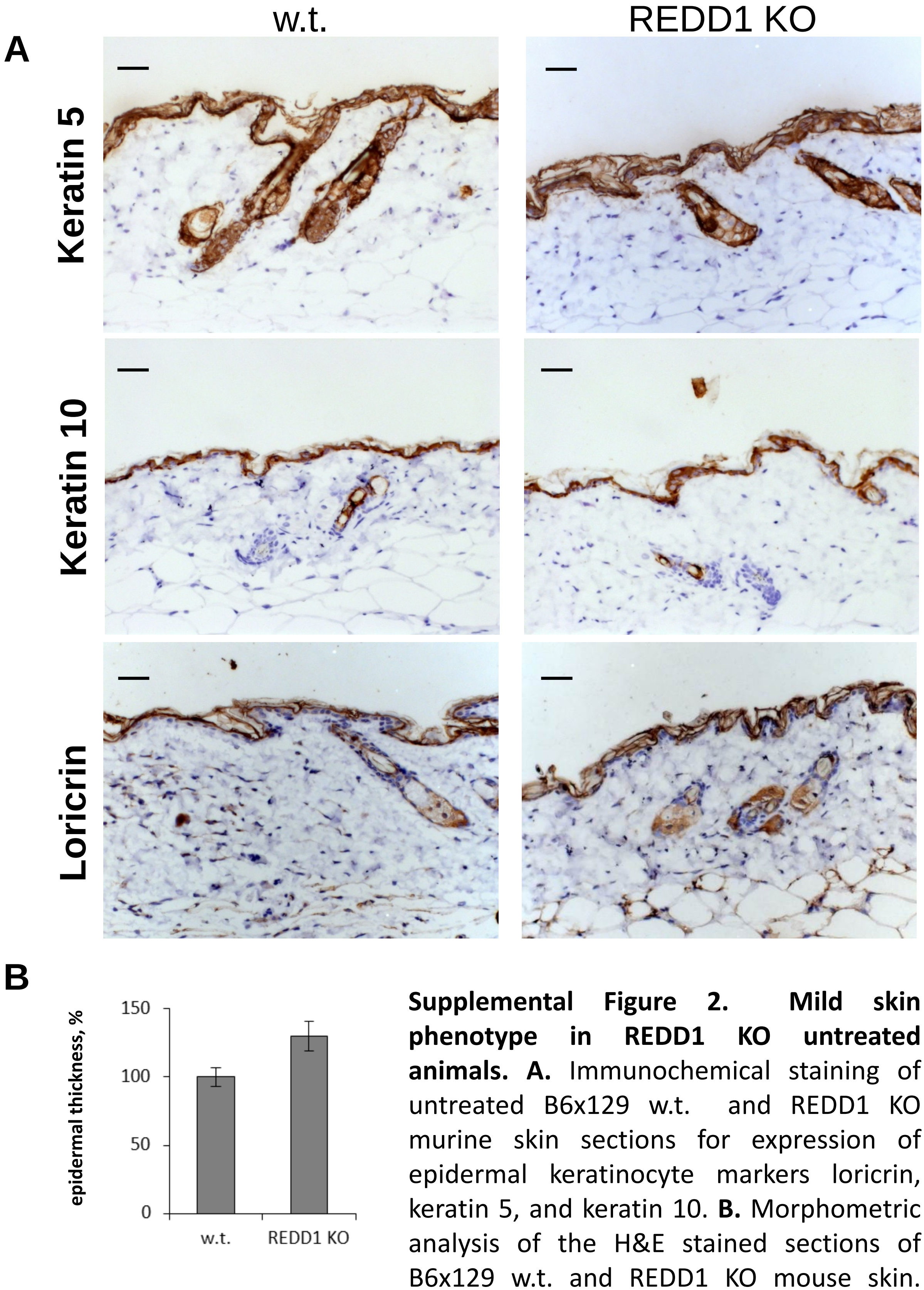

Supplemental Figure 2. Mild skin phenotype in REDD1 KO untreated animals. A. Immunochemical staining of untreated B6x129 w.t. and REDD1 KO murine skin sections for expression of epidermal keratinocyte markers loricrin, keratin 5, and keratin 10. **B.** Morphometric analysis of the H&E stained sections of B6x129 w.t. and REDD1 KO mouse skin. REDD1 KO skin thickness is presented as % to thickness of w.t. epidermis.
