# Supplementary material for: REDD1 functions at the crossroads between the therapeutic and adverse effects of topical glucocorticoids
Source: EMBO Mol Med. 2014 Dec 11;7(1):42–58. doi: 10.15252/emmm.201404601 (PMC4309667; doi:10.15252/emmm.201404601)
Supplement: Supplementary file 3 [file emmm0007-0042-sd3.pdf]

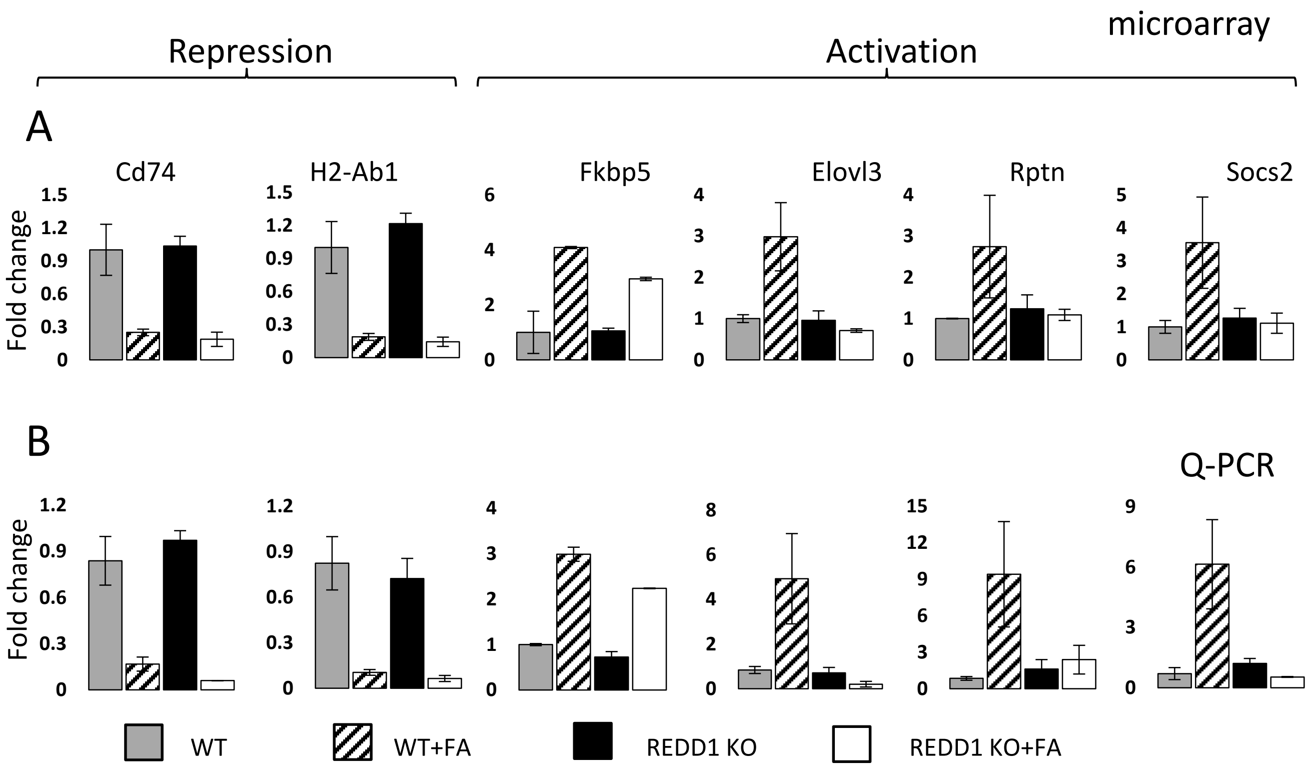

C

**PCC**	0.99	0.96	0.97	0.98	0.98	0.99

Supplemental Fig. 3. Validation of microarray data by Q-PCR. Six genes from different GO groups representing typical pattern of glucocorticoid-induced differential gene expression in epidermis in w.t. and REDD1 KO milieu were selected for validation. **A.** Microarray data for the selected genes. **B.** Q-PCR data for the selected genes. **C.** Pearson correlation coefficients (PCC) between microarray and Q-PCR data for the corresponding genes. Bars represent mean ± SD calculated for two individual skin samples.
