# Supplementary figures and images for: REDD1 functions at the crossroads between the therapeutic and adverse effects of topical glucocorticoids
Source: EMBO Mol Med. 2014 Dec 11;7(1):42–58. doi: 10.15252/emmm.201404601 (PMC4309667; doi:10.15252/emmm.201404601)

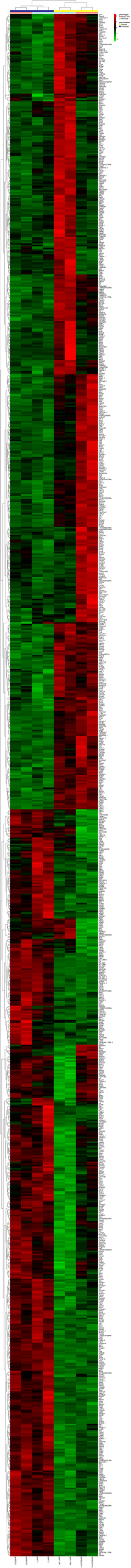

Supplement: Supplementary file 4 [file emmm0007-0042-sd4.pdf]
