# Supplementary material for: REDD1 functions at the crossroads between the therapeutic and adverse effects of topical glucocorticoids
Source: EMBO Mol Med. 2014 Dec 11;7(1):42–58. doi: 10.15252/emmm.201404601 (PMC4309667; doi:10.15252/emmm.201404601)
Supplement: Supplementary file 5 [file emmm0007-0042-sd5.pdf]

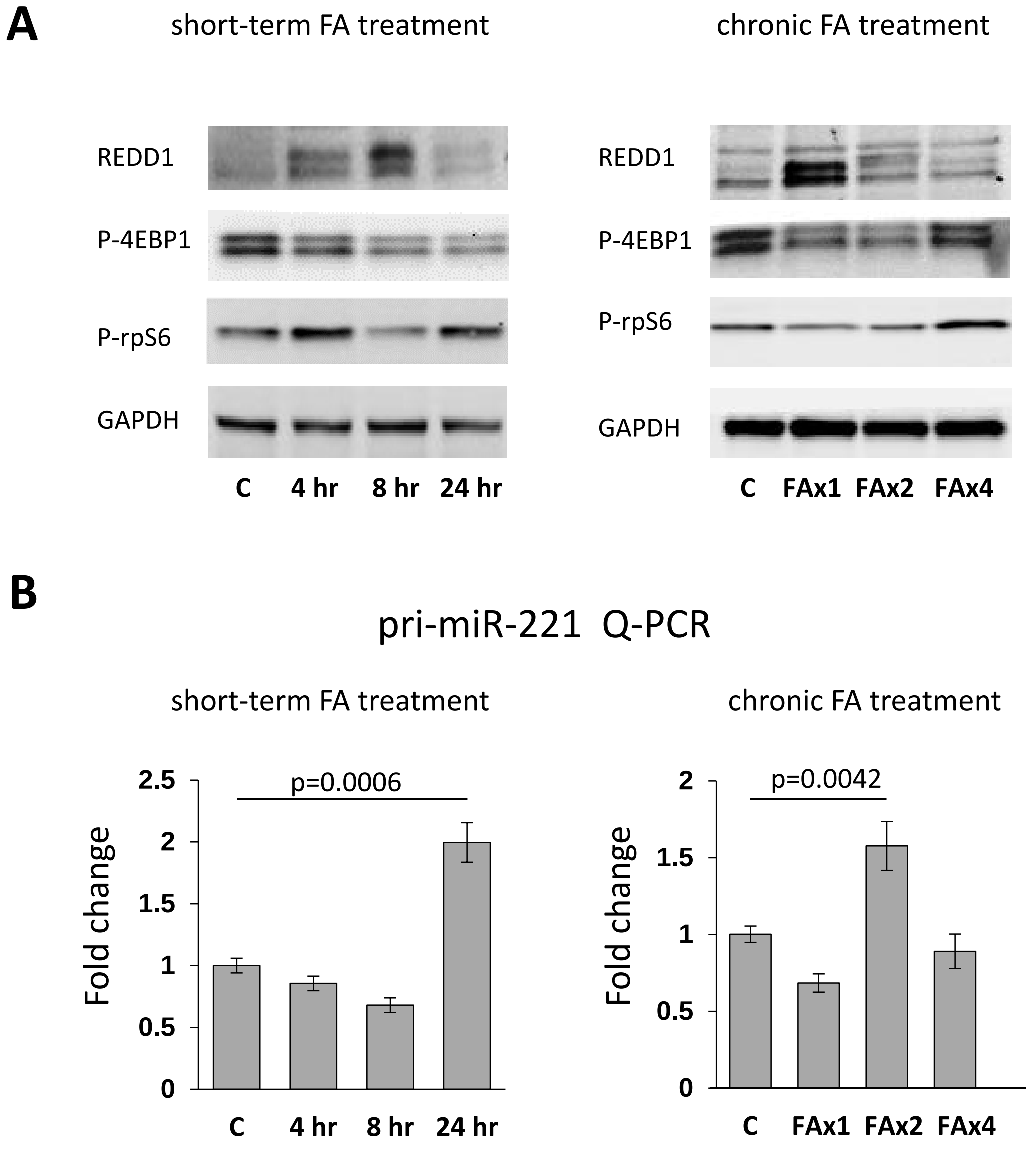

Supplemental Figure 5. Inverse correlation between pri-miR-221 and REDD1 expression during glucocorticoid treatment. B6D2 mice were treated with FA topically as described in Materials and Methods, epidermis was harvested at indicated time points. **A.** Western blot analysis. **B.** Q-PCR analysis of primary miR-221. Data are expressed as the fold change (mean ± SD) with respect to the controls (unpaired two-tailed t-test, n = 3).
