# Supplementary material for: REDD1 functions at the crossroads between the therapeutic and adverse effects of topical glucocorticoids
Source: EMBO Mol Med. 2014 Dec 11;7(1):42–58. doi: 10.15252/emmm.201404601 (PMC4309667; doi:10.15252/emmm.201404601)
Supplement: Supplementary file 6 [file emmm0007-0042-sd6.pdf]

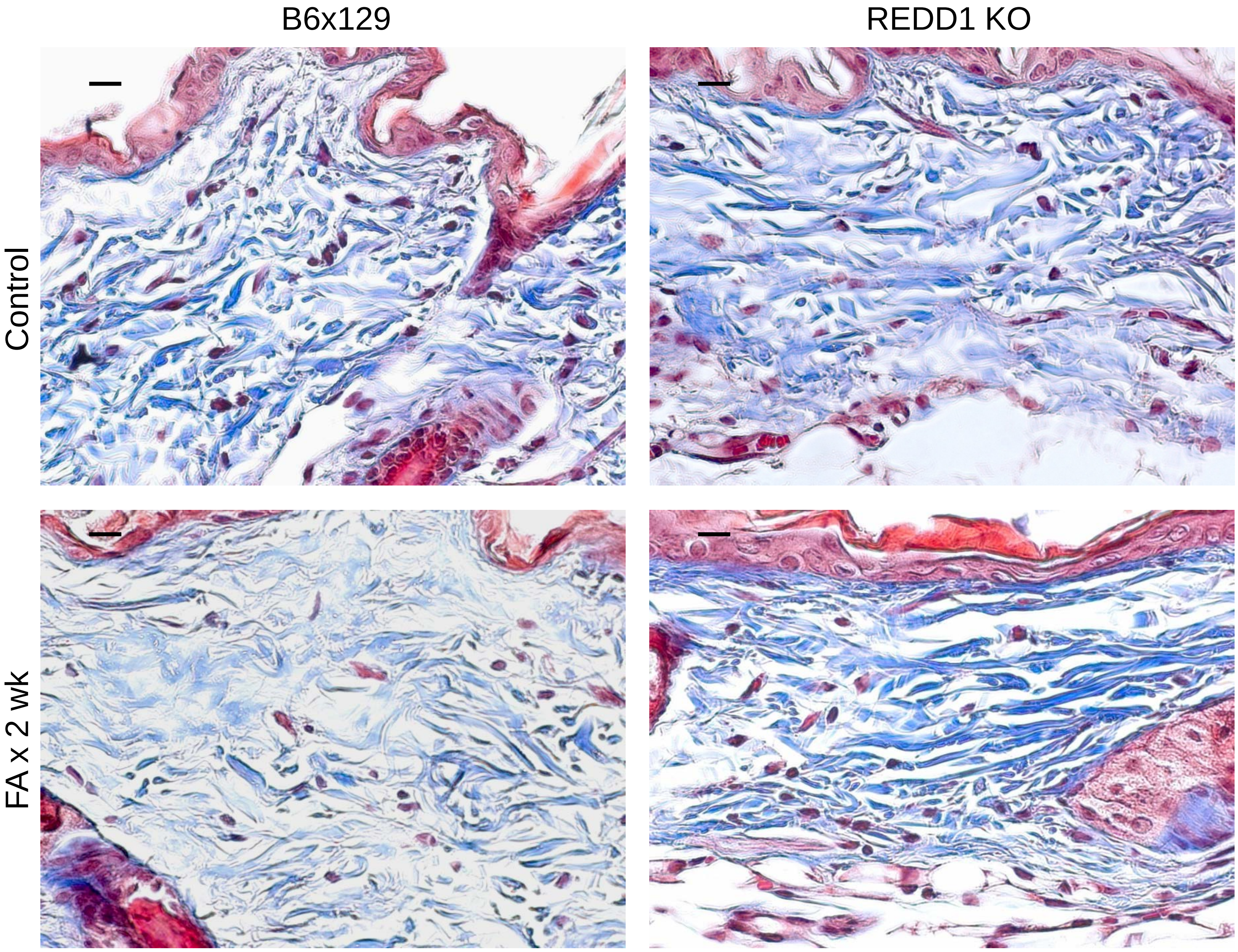

Supplemental Figure 6. Resistance of REDD1 KO mice to glucocorticoid-induced decrease of dermal cellularity. REDD1 KO and isogenic w.t. B6x129 mice were treated with acetone (vehicle control) or FA (2 µg/animal) every 72 hr for 2 wk. Formalin-fixed paraffin-embedded skin sections were used for Masson's trichrome staining. Dermis/collagen fibers are blue, muscle is red, nuclei are dark red, and cytoplasm is red/pink. Scale bars are 40 µm. **Note**: strongly decreased number of dermal fibrobalsts in the skin of w.t. mice treated with FA x 2 wk.
