# Supplementary material for: REDD1 functions at the crossroads between the therapeutic and adverse effects of topical glucocorticoids
Source: EMBO Mol Med. 2014 Dec 11;7(1):42–58. doi: 10.15252/emmm.201404601 (PMC4309667; doi:10.15252/emmm.201404601)
Supplement: Supplementary file 9 [file emmm0007-0042-sd9.doc]

**Supplemental Table 3. Most enriched Gene Ontology (GO) categories, fold enrichment cutoff ≥ 3.0, P<0.01**

**w.t.: FA vs control, up-regulated genes**

| **Term** | **P-value** | **Fold Enrichment** |
| --- | --- | --- |
| **GO:0006665~sphingolipid metabolic process** | 3.85E-04 | 5.87 |
| **GO:0006643~membrane lipid metabolic process** | 4.77E-04 | 5.67 |
| **GO:0004091~carboxylesterase activity** | 8.59E-04 | 4.48 |
| **GO:0006672~ceramide metabolic process** | 1.17E-03 | 7.39 |
| **GO:0046519~sphingoid metabolic process** | 1.52E-03 | 6.98 |
| **GO:0006644~phospholipid metabolic process** | 3.28E-03 | 3.05 |
| **GO:0016765~transferase activity, transferring alkyl or aryl (other than methyl) groups** | 3.75E-03 | 5.71 |
| **GO:0046479~glycosphingolipid catabolic process** | 5.39E-03 | 25.11 |
| **GO:0005885~Arp2/3 protein complex** | 5.75E-03 | 24.28 |

**REDD1 KO: FA vs control, up-regulated genes**

| **Term** | **P-value** | **Fold Enrichment** |
| --- | --- | --- |
| **GO:0005776~autophagic vacuole** | 4.99E-04 | 23.64 |
| **GO:0051270~regulation of cell motion** | 5.20E-03 | 3.78 |
| **GO:0042598~vesicular fraction** | 5.84E-03 | 3.02 |
| **GO:0005770~late endosome** | 6.43E-03 | 6.65 |
| **GO:0008440~inositol trisphosphate 3-kinase activity** | 8.09E-03 | 20.98 |
| **GO:0030334~regulation of cell migration** | 9.17E-03 | 3.88 |

**w.t.: FA vs control, down-regulated genes**

| **Term** | **P-value** | **Fold Enrichment** |
| --- | --- | --- |
| **GO:0006260~DNA replication** | 1.35E-06 | 4.72 |
| **GO:0002495~antigen processing and presentation of peptide antigen via MHC class II** | 3.00E-05 | 15.11 |
| **GO:0019886~antigen processing and presentation of exogenous peptide antigen via MHC class II** | 3.00E-05 | 15.11 |
| **GO:0042613~MHC class II protein complex** | 4.99E-05 | 21.39 |
| **GO:0002504~antigen processing and presentation of peptide or polysaccharide antigen via MHC class II** | 5.91E-05 | 13.33 |
| **GO:0002478~antigen processing and presentation of exogenous peptide antigen** | 2.26E-04 | 10.30 |
| **GO:0019882~antigen processing and presentation** | 3.27E-04 | 5.15 |
| **GO:0005773~vacuole** | 4.65E-04 | 3.01 |
| **GO:0019884~antigen processing and presentation of exogenous antigen** | 6.21E-04 | 8.39 |
| **GO:0048002~antigen processing and presentation of peptide antigen** | 1.39E-03 | 7.08 |
| **GO:0042287~MHC protein binding** | 1.87E-03 | 15.19 |
| **GO:0030145~manganese ion binding** | 2.35E-03 | 3.19 |
| **GO:0005657~replication fork** | 3.10E-03 | 8.02 |
| **GO:0042611~MHC protein complex** | 3.51E-03 | 5.78 |
| **GO:0005663~DNA replication factor C complex** | 3.87E-03 | 28.88 |

**REDD1 KO: FA vs control, down-regulated genes**

| **Term** | **P-value** | **Fold Enrichment** |
| --- | --- | --- |
| **GO:0002495~antigen processing and presentation of peptide antigen via MHC class II** | 8.11E-06 | 19.77 |
| **GO:0019886~antigen processing and presentation of exogenous peptide antigen via MHC class II** | 8.11E-06 | 19.77 |
| **GO:0003735~structural constituent of ribosome** | 1.44E-05 | 4.85 |
| **GO:0002504~antigen processing and presentation of peptide or polysaccharide antigen via MHC class II** | 1.62E-05 | 17.45 |
| **GO:0042613~MHC class II protein complex** | 2.74E-05 | 24.92 |
| **GO:0002478~antigen processing and presentation of exogenous peptide antigen** | 6.34E-05 | 13.48 |
| **GO:0019884~antigen processing and presentation of exogenous antigen** | 1.79E-04 | 10.98 |
| **GO:0006631~fatty acid metabolic process** | 1.98E-04 | 3.71 |
| **GO:0019882~antigen processing and presentation** | 3.50E-04 | 5.99 |
| **GO:0005840~ribosome** | 3.64E-04 | 3.47 |
| **GO:0048037~cofactor binding** | 3.70E-04 | 3.26 |
| **GO:0048002~antigen processing and presentation of peptide antigen** | 4.11E-04 | 9.27 |
| **GO:0033559~unsaturated fatty acid metabolic process** | 5.50E-04 | 8.72 |
| **GO:0042287~MHC protein binding** | 9.42E-04 | 19.26 |
| **GO:0016126~sterol biosynthetic process** | 1.70E-03 | 9.51 |
| **GO:0042611~MHC protein complex** | 1.81E-03 | 6.73 |
