# Supplementary material for: REDD1 functions at the crossroads between the therapeutic and adverse effects of topical glucocorticoids
Source: EMBO Mol Med. 2014 Dec 11;7(1):42–58. doi: 10.15252/emmm.201404601 (PMC4309667; doi:10.15252/emmm.201404601)
Supplement: Supplementary file 10 [file emmm0007-0042-sd10.doc]

**Supplemental Table 4**

**Primer sets for Quantitative and Semi-quantitative RT-PCR analysis**

| Gene symbol | | Primer sequence: sense/antisense (5'-3') |
| --- | --- | --- |
| mouse | human |  |
| REDD1 |  | GGG CCG GAG GAA GAC TCC TCA TA  CTG TAT GCC AGG CGC AGG AGT TC |
| GR |  | CGC AGG CCG CTC AGT GTT TTC TA  TGT GCT GTC CTT CCA CTG CTC TT |
| Krt5 |  | GCG GGA GTA CCA GGA GCT CAT GA  AAG CCA CTG CCA ACA CCA ATG CT |
| Adipoq |  | | AGA GAA AGG AGA TGC AGG TCT TC | | | --- | --- | | TAA AGC GAA TGG GTA CAT TGG A |  | |
| Ivl |  | TCC CTC CTG TGA GTT TGT TTG GTC T  TCT CCT CAT GTT TGG GAA AGC CCT |
| Lep |  | CTG CAA GGT GCA AGA AGA AGA AG |
|  |  | GAA TGA AGT CCA AGC CAG TGA C |
| H2-Ab1 |  | ACA TCT ACA ACC GGG AGG AGT A |
|  |  | GAC GAC ATT GGG CTG TTC AAG |
| Fkbp5 |  | GGT TTT GGA GAA GCC GGG AAG CC |
|  |  | CCT GCG TGT ACT TGC CTC CCT TG |
| Elovl3 |  | TTT TGG AGG AGT ACT GGG TAA GC  CCA CTG TAA ACA TCA CTG TTG CC |
| Hilpda |  | CGT GCA GGA TCT AGC AGC AGA A  CTT AGG AGG CTG TGT GTT GGC TA |
| Socs2 |  | TCA GTC AAA CAG GAT GGT ACT GG  TGT TTA AGC TTG GAC TTG ACA CA |
| Rptn |  | GAG ACA TCC TTC GGA GAC CAA AT  ATC ATG TTC TTT CTG GGA GGA GG |
| Cd74 |  | GCG ACG AGA ACG GTA ACT ATT TG  CTC TGT CTT CAC AGG GTG ACT TG |
| pri-miR-221 |  | GCT GTT GCC TAA CAA ACA CAG AA  GCA TGA TGA ATG ACC ACC AAT CG |
| Rpl27 |  | GCC CTG GTG GCT GGA ATT GAC C  TTG CGC TTC AAA GCT GGG TCC C |
|  | REDD1 | TAG CCT TTG GGA CCG CTT CTC GT  CAG GTA AGC CGT GTC TTC CTC CG |
|  | RPL27 | ACC GCT ACC CCC GCA AAG TG  CCC GTC GGG CCT TGC GTT TA |
