# Supplementary material for: REDD1 functions at the crossroads between the therapeutic and adverse effects of topical glucocorticoids
Source: EMBO Mol Med. 2014 Dec 11;7(1):42–58. doi: 10.15252/emmm.201404601 (PMC4309667; doi:10.15252/emmm.201404601)
Supplement: Supplementary file 11 [file emmm0007-0042-sd11.pdf]

EMBO Molecular Medicine

REDD1 functions at the crossroads between the therapeutic and adverse effects of topical glucocorticoids

Gleb Baida, Pankaj Bhalla, Kirill Kirsanov, Ekaterina Lesovaya, Marianna Yakubovskaya, Kit Yuen, Shuchi Guo, Robert M Lavker, Ben Readhead, Joel T Dudley, Irina Budunova

Corresponding author: Irina Budunova, Feinberg School of Medicine, Northwestern University

---

Review timeline:

Submission date:	26 September 2013
Editorial Decision:	30 September 2013
Appeal:	14 October 2013
Editorial Decision:	17 December 2013
Additional Author correspondence:	23 December 2013
Additional Editorial Corrrespondence:	27 December 2013
Resubmission:	03 September 2014
Editorial Decision:	26 September 2014
Revision received:	16 October 2014
Editorial Decision:	28 October 2014
Revision received:	29 October 2014
Accepted:	30 October 2014

Transaction Report:

(Note: With the exception of the correction of typographical or spelling errors that could be a source of ambiguity, letters and reports are not edited. The original formatting of letters and referee reports may not be reflected in this compilation.)

Editor: Roberto Buccione

1st Editorial Decision 30 September 2013

Thank you for the submission of your manuscript "REDD1 (regulated in development and DNA damage response 1) dissociates therapeutic and adverse effects of topical glucocorticoids in skin".

I have now had the opportunity to carefully read your paper and the related literature and I have also discussed it with my colleagues. I am afraid that we concluded that the manuscript is not well suited for publication in EMBO Molecular Medicine and have therefore decided not to proceed with peer review.

You find REDD1 among the most up-regulated genes after glucocorticoid (GC)-treatment of mouse and human skin and that in the former, autophagy increased up to 8 hrs after treatment together with concomitant maximal REDD1 expression and mTOR inhibition. You also find that REDD1 KO mice appear to be more resistant to GC-induced skin atrophy without loss of therapeutic efficacy. We appreciate that REDD1 did not appear to affect GC receptor protein levels in skin but modified receptor function by shifting it towards transrepression.

Although we acknowledge the potential interest of your findings, due also to the available knowledge, we are not persuaded that your manuscript provides the striking conceptual advance, mechanistic insight and (novel) translational implications we would like to see in an EMBO

Molecular Medicine article. As such, your manuscript appears better suited to a specialistic venue.

I am sorry that I could not bring better news.

Appeal 14 October 2013

I am thankful to you for the decision letter with a personal touch. And I would like to respond to your letter.

I was obviously upset as we thought that the paper should be of interest for EMBO Molecular Medicine readers. But most of all I was surprised by your conclusion that the major weaknesses are the lack of novelty and novel translational implications. I study the glucocorticoid receptor for @ 20 years, and I have not seen the attempts to find out the direct role of GR target genes in glucocorticoid therapeutic and side effects.

This is true that REDD1 induction by glucocorticoids is known, and steroid atrophic effect in muscle (muscle waste) has been linked to REDD1. However, we went much further.

1. We discovered that REDD1 is induced in skin by steroids and used REDD1 KO animals (as far as I know for the first time) to directly prove the role of REDD1 as atrophogenic gene in skin. Importantly, we were able to prove that molecular mechanisms behind steroid-induced atrophy in muscle, skin and adipose are remarkably similar at the global genomic level. We also found that REDD1 is a leading gene in atrophy as assessed by its "molecular weighted contribution". We are working with the leading American experts in biostatistics and meta-array analysis, and can easily provide the additional bioinformatics data to strengthen our conclusions.

2. We found (and it was an unexpected and striking discovery) that REDD1 can dissociate good and bad effects of glucocorticoids in skin, and potentially in other organs/tissues as the molecular mechanisms of steroid-induced atrophy are so similar according to our data. These results serve as a foundation for US patent for novel approach to reduce side effects of glucocorticoids using REDD1 inhibitors.

3. We also discovered (and it was unexpected too) that REDD1 modifies GR function in a very interesting and unique way: in the absence of REDD1 only one branch of GR activity- activation of gene expression suffers. At the same time, GR transrepression (negative regulation of GR target gene expression) remains almost intact. This is absolutely novel finding that can mechanistically explain the dissociation of therapeutic and adverse effects of glucocorticoids in the absence of REDD1.

I agree that it would be important to know more regarding specific steps in GR activation (phosphorylation, dimerization, nuclear translocation, loading at the gene promoters) that are affected by REDD1. We are planning to do this in the nearest future, but it is whole new level of research, and will require a very significant time and effort to identify these mechanisms.

In conclusion, I would be very thankful if you could let me know whether and how we can improve our manuscript to allow you to reconsider your decision.

Thank you very much for your consideration in advance,

2nd Editorial Decision 17 December 2013

Thank you for the submission of your manuscript to EMBO Molecular Medicine. We have now heard back from the three referees whom we asked to evaluate your manuscript.

We are very sorry that it has taken much longer than usual to get back to you on your manuscript. In

this case we experienced significant difficulties in securing three expert and willing Reviewers. Further to this, one evaluation was delivered with considerable delay.

As you will see, while Reviewer 1 appears more supportive of your study, Reviewers 2 and 3 point to significant and fundamental issues that, I am afraid, preclude publication of the manuscript in EMBO Molecular Medicine. I will not discuss each point in detail as they are clearly stated. There are, however, some crucial points, in many cases shared by the Reviewers, that I wish to bring to your attention.

Reviewer 1 considers the data presentation and analysis to be superficial - especially relating to the expression analysis. Reviewer 1 also mentions other issues.

Reviewer 2 notes the limited novelty and that the data do not clarify/explain the transient effects of glucocorticoid treatment on REDD1 protein expression, mTORC1 signaling, and autophagy markers, and the sustained loss of tissue, and the loss REDD1 protein in the presence of sustained REDD1 mRNA expression.

Reviewer 3 is especially critical and has many concerns that ultimately point to lack of sufficient support, experimental flaws and inadequate presentation. This Reviewer also notes the limited conceptual advance and overall novelty and finds that the clinical impact is unclear at this stage (quite a relevant aspect for EMBO Molecular Medicine). Reviewer 3's critique is detailed, documented and thorough and we agree with his/her assessment.

I would also like to point out that all three Reviewers lament the fact that the full gene expression dataset was not shared.

Given these fundamental concerns and the overall lack of enthusiasm by the Reviewers, I have no choice but to return the manuscript to you at this stage. In our assessment it is not realistic to expect to be able to address these issues experimentally in a reasonable time frame and to the satisfaction of the Reviewers.

I am sorry to have to disappoint you at this stage. I hope that the Reviewers' comments will be helpful in your continued work in this area.

***** Reviewer's comments *****

Referee #1 (Comments on Novelty/Model System):

The data are not well analysed in terms of objective assessment. They are interesting and I think with a more thorough analysis they have something of real significance. I particularly found the gene expression profiling data to be very basic and the constant use of RT-PCR for relative expression analysis is outdated.

Referee #1 (Remarks):

There has been a lot of interest in area of glucocorticoids in wound healing - clinically a very important area. This study identifies a potential mechanism that underlies skin atrophy, and shows that it is distinct from the mechanism from that which mediates GC anti-inflammatory effects. The real strength of the study comes from the REDD1 null mouse approach, and the histological data are convincing. I think their findings are interesting but the analysis comes across as rather superficial - particularly relating to the expression analysis. It is barely mentioned in the M+M and there is no detail regarding public availability of data.

I have a number of comments going through the paper as written, as detailed below.

Furthermore the data are already published as an abstract, but I don't know whether this impacts on your decision:

Journal of Investigative Dermatology (2013) 133, S243-S246. doi:10.1038/jid.2013.106
Deletion of mTOR inhibitor REDD1 protects CD34+ follicular epithelial stem cells and prevents development of steroid-induced cutaneous atrophy
G Baida, P Bhalla, K Yuen, S Guo, RM Lavker and I Budunova

P5 penultimate paragraph. "glucocorticoid-induced muscle wasting,...

P7 2nd para. RT-PCR data are not truly quantitative, so the values listed don't really mean anything.

Fig1
Sets up the study nicely

P7 last sentence - is it mega or meta-analysis? I thought meta.

P8 If they are going to address REDD1 and induction of autophagy, they really need to reference prior work - see Molitoris 2011 (below)

Fig2
B. Rpl27 isn't a real control for miRNA RT-PCR (which isn't quantitative in any case - see above).
C. What is their positive control for bortezomib treatment?
D. Why don't they also quantitate Beclin 1 - the Beclin western doesn't look too convincing.

P8 I think the last sentence would be more logical, given Fig 2B.1 data, and clearer to the reader if they said "increase in TXNIP expression may contribute to REDD1 stabilization"

Fig3
A+B don't really convey useful information - the FACS data are sufficient and also objective.

Fig4
C. P63 IHC isn't very well presented - counterstain too strong and difficult for reader to discern positive nuclear staining.
Otherwise the data are very clear in this figure and suggest that there is no significant effect on anti-inflammatory effects.

Fig5
Looking at the difference in epidermal thickness in Fig5B it would be good to really clarify in Fig 4B that the untreated controls are both set at 100% - why don't they just show relative to wt control? That would be a more informative representation and wouldn't change their findings. Is the difference in thickness statistically significant? I don't really see any value in this figure - it could be supplementary, if it is even needed. The data in 5B could easily be incorporated in Fig 4 as detailed above. This is corroborated by the fact they barely refer to the figure in the results.

Fig6
What do they mean by a modest increase in GR expression - certainly not at mRNA level (although this isn't quantitative) and where are the quantitative data for the protein level? Seems rather anecdotal.
The bioinformatics analysis is rather odd - they look at the 100 most up/down-regulated genes in wt mice, and then compare what happens in REDD1 null mice. However, they don't say how many genes are differentially regulated in the null mice, and how closely the wt top 100 selections mirror the null top 100 selections. It all seems rather superficial and lacking in true bioinformatic interrogation.
Furthermore, why are there 4 boxes in 6B but only 3 descriptions.
On what basis were the genes in C selected? Were they the top hits?
D. The significant increase in FA treatment of REDD1 overexpressing cells is distinctly underwhelming for a luciferase assay - in my experience these data are typically far more dramatic when reflecting real biological effects. A difference of maybe 20% between the two is not convincing to me.

Are they making their bioinformatics data available?

They could discuss whether REDD1 is druggable.

Missing reference
J Biol Chem. 2011;286(34):30181-9. Glucocorticoid elevation of dexamethasone-induced gene 2 (Dig2/RTP801/REDD1) protein mediates autophagy in lymphocytes. Molitoris JK, McColl KS, Swerdlow S, Matsuyama M, Lam M, Finkel TH, Matsuyama S, Distelhorst CW.

Referee #2 (Comments on Novelty/Model System):

This is not a particularly well written manuscript and would need a considerable amount of editorial work before it would be suitable for publication. In particular, the "story line" rambles through an analysis of the various effects of glucocorticoids that have been observed in other tissues. It finally gets to Figure 6 which represents the truly novel aspects of the work. Unfortunately, the authors choose not to share all the results of the gene expression analysis. These results would be of paramount interest to readers of the journal. In my opinion, further consideration of the manuscript would require inclusion of all the results.

Referee #2 (Remarks):

The results presented in this manuscript show that glucocorticoids act to increase expression of REDD1 in mouse and human epidermis as the hormones have previously been shown to do in other tissues including muscle and adipose tissue. As expected, based on previous studies, the increased expression of REDD1 is accompanied by skin atrophy, repressed mTORC1 signaling, increased autophagy, and reduced cell growth. The results also suggest that REDD1 knockout mice might be resistant to glucocorticoid-induced atrophy of skin but retain sensitivity to the anti-inflammatory effects of glucocorticoids.
1. It is not clear to the reviewer how to reconcile the rather transient effects of glucocorticoid treatment on REDD1 protein expression, mTORC1 signaling, and autophagy markers, i.e., maximal effect at 8 hours and return to control values by 24 hours (Fig 2), with the sustained loss of tissue. Also not clear is the basis for the loss of REDD1 protein in the face of a sustained REDD1 mRNA expression. The authors attempt to address this point through the use of a proteasome inhibitor, which had no effect; however, no data is shown for a positive control for the inhibitor. Since REDD1 protein has been reported to be degraded through a proteasome-dependent mechanism, the basis for these negative results is unclear.
2. Rationale not clear for choice of P-rpS6 and mTOR (Ser 2448 - an AKT site) as biomarkers of mTORC1 signaling rather than the more typical markers such as p70S6K (Thr 389) and mTOR (Ser 2481). Concern is that rpS6 is a target for other signaling pathways.
3. Not sure if Figure 3 adds much value to main point of manuscript; likewise for miR-221.
4. Are glucocorticoid treatment groups in Fig 4B significantly different?
5. Figure 6 represents the novel findings reported in the manuscript. Authors should present all the gene expression data.

Referee #3 (Comments on Novelty/Model System):

Technical quality of this manuscript is poor in part due to experimental design and in part because of low quality.

The quality of representative images for histology, immunohistochemistry stainings are not convincing and do not support conclusions. Specifically, Figures 1C1, 4C and 4D. Image in Figure

1C1 is cut off at the corners of rete ridges. It is very difficult to base conclusions solely on images and quantification of BrDU is necessary in Figure 4C. Similarly, showing FACS data for quantification of CD34 cells would be more convincing. Such difference in CD34 staining, as presented in Fig 4d, may imply differences in number of hair follicles, which should be addressed.

Based on the data presented in Fig 1D1 scraping method does not show adequate purity of fat fraction (contamination with epidermal markers (K5 and involucrin) is visible on Western blot). Enzymatic digestion and cell sorting should be used instead.

Choice of 3PC cell line instead of primary mouse and human keratinocytes are not justifiable and could be misleading. In addition, based on the images shown it appears that confluence and culture conditions are different between control and REDD1.

Quantification of PCR is questionable throughout the manuscript. For example, the authors have chosen to quantify RT- PCR results using indirect method and gel electrophoresis instead of using real time PCR which might be misleading, such as in a case of miR-221 (Figure 2B2). Please provide details on PCR cycle numbers. Quantifying miRNA using a regular transcript for normalization (Rpl27) is not fully appropriate. Please provide qPCR with using small RNA expression as a house-keeping gene.

REDD1-mediated atrophy by glucocorticoids has been reported reported in muscle tissue. Here authors expand on similar mechanism in skin but fail to present convincing evidence.

Clinical impact is unclear since the effects on human skin differ from mouse (even in the only experiment shown in this paper). Majority of data are collected from mouse in vivo or mouse cells including cell lines rather than primary cells. Correlation with human cells and tissue is essential for any potential clinical interpretation since in many cases translation from mouse failed to replicate human condition.

Referee #3 (Remarks):

The manuscript " REDD1 dissociates therapeutic and adverse effect of topical glucocorticoids in skin" by Baida G et al describes REDD1-mediated mechanisms of glucocorticoids in skin through mTOR pathway. REDD1 has already been identified as a GR target gene in muscular tissue. Authors have confirmed this in mouse and human skin treated with glucocorticoids and proceed to use REDD1 knockout to show partial protection from GR atrophy in epidermis and fat tissues. REDD1 KO remained sensitive to anti-inflammatory effects of glucocorticoids, suggesting that REDD1 dissociates beneficial for the adverse effects of glucocorticoids in skin. Although interesting topic that may potentially have clinical impact in the future the quality of data, experimental design and methods limit overall enthusiasm.

There are several issues that need to be considered and addressed. Glucocorticoid action is not fully addressed at several levels. Confirmation of GR activation was not assessed. Given experimental design with various treatment protocols (and compunds) levels of phosphorylated GR was not measured. Furthermore, distinction between non-genomic and genomic effects is necessary in the context of distinguishing between various effects of glucocorticoids. Finally, endogenous cortisol activity and enzymes that regulate it (HSD11b1/2) were not even acknowledged and can have important influence on the data outcome.

Remarks regarding novelty, and technical quality are outlined above. In addition, data shown in Figure 6 are not informative. Microarray analyses that claims 50% difference is not documented. Pie charts do not support any conclusion since no % or number of genes are presented and should be included. Additional table listing top regulated genes should also be included and complete data sets submitted according to guidelines.

Fat and epidermis are not only tissue components to which side effects of glucocorticoids can be attributed. Skin atrophy includes major effects on dermal component which was not included. Such data are necessary to support the conclusions of distinction between anti-inflammatory effects and skin atrophy.

Additional Author correspondence 23 December 2013

I am thankful for your detailed letter and for the helpful analysis of our work by reviewers.
I looked very carefully at the reviewers' comments and questions, and I agree with many of them.
I would like to let you know that during last two and a half months, since manuscript submission to your journal, we have been actively working on the REDD1 project.
Thus, we are now in a very good position to address the reviewers' comments and questions.

Specifically,

- We are in the process of detailed DNA array analyses (this is the major comment made by all reviewers). This is in collaboration with Dr. Dudley, Director of Biomedical Informatics, Mount Sinai School of Medicine (New York, USA).
- The results of semi-quantitative RT-PCR have been already repeated and quantified by image analysis (another major comment made by all reviewers).
- We have been working with primary human keratinocytes to confirm the results obtained in mice and to make steps towards clinical translation of our findings.
- We have already assessed the specific stages in the activation of the glucocorticoid receptor (GR phosphorylation and nuclear translocation) that are affected by REDD1 expression status in keratinocyte cell lines. We will repeat these experiments using primary human keratinocytes.

I would like to acknowledge that our work with primary human keratinocytes is supported by Northwestern University SDRC (Skin Disease Research Center), and its Cores that provide human keratinocytes in 2D cultures as well as keratinocyte rafts (organotypic, 3D cultures).

After the careful assessment of the new results that we have already obtained, and the additional experiments that we will need to perform to respond to reviewers' comments, I feel confident that we will be able to do it in a reasonable time frame. However, this may involve considerable changes in the manuscript. Thus, I would be thankful for your advice whether to submit the manuscript as revised or as a new manuscript to your journal.

Thank you very much for your consideration in advance.

Additional Editorial Corrrespondence 27 December 2013

Thank you for your detailed note and apologies for not being able to reply sooner.

I definitely suggest that you submit an extensively revised manuscript as a de novo submission. I would ask you, however, to also submit a detailed point by point rebuttal to help me and the potential Reviewers in evaluating the new submission.

Resubmission 03 September 2014

Response to specific comments.

Referee #1

- *The data have been published as an abstract.*

- The publication of preliminary data as abstracts is not prohibitive for publication of study as a research paper at EMBO Mol Medicine.

- *The "analysis comes across as ...superficial - particularly .. the expression analysis".*

- In-depth analysis of expression profiling studies was performed by experts and is described in "general comments"; the data are submitted to NCBI.

- *RT-PCR data are not truly quantitative.*

- We have replaced all these with real-time PCR (Q-PCR). Pearson correlation analysis shows perfect correlation between array data and Q-PCR results (Supplemental Figure 3). As we were limited by sample availability from healthy volunteers, we were unable to measure REDD1 message in human skin by Q-PCR (Fig. 1).

- *In Fig. 2 Rpl27 isn't a real control for miRNA RT-PCR.*

- In revised manuscript, we evaluated pri-miR-221 levels by Q-PCR. Rpl27 is a proper normalization control for precursors of microRNA, because both microRNA and mRNA are synthesized by RNA polymerase II. We found a significant increase in pri-miR-221 at the time points when REDD1 levels start declining (Supplemental Fig. 5) suggesting that mature miR-221 may be involved in control of REDD1 induction by steroids in skin.

- What was the positive control for bortezomib treatment?

- This resonates with the comments of Reviewer #2 ("REDD1 protein has been reported to be degraded by proteasome-dependent mechanism, thu the … "results with bortezomib are unclear") suggesting that suboptimal design of our experiment to assess REDD1 stability during chronic glucocorticoid treatment causing difficulties for the data interpretation. We thus removed this part of the study from the paper.

- *Quantify the expression of autophagy marker, Beclin-1.*

- Western blots for Beclin-1 were quantified and are presented in Fig. 2D.

- *Figs. 3A and B do not convey useful information.*

- These figures (the effect of REDD1 on keratinocyte size) were eliminated.

- *p63 immunostaining is of insufficient quality.*

- We repeated p63 IHC, and quantified p63+ progenitors in the epidermis (modified Fig. 4 A-B). This analysis confirms the protection of skin stem cells/ progenitors by the absence of REDD1.

- *The reviewer suggested report the increased baseline epidermal thickness in REDD1 KO (previously Fig. 5) as we describe their resistance to steroid-induced skin atrophy.*

- We followed this advice, and made suggested changes (now Fig. 3C).

- The data from the figure previously numbered as Fig 5 (mild skin phenotype in REDD1 KO) are relegated to supplementary Fig. 2.

- *Insufficient data on GR expression in REDD1-KO epidermis.*

- We repeated Western blot analysis of GR expression in REDD1 KO epidermis (now Fig. 4D), ORCs and HaCaTs after REDD1 shRNA knockdown (new Figs. 5 and 7). In all cases the knockout/knockdown of REDD1 caused no decrease in GR expression suggesting that changes in GR function were not caused by its diminished expression.

- All critiques related to the bioinformatics analysis have been addressed (see above).

- *The changes in activity of GRE.Luciferase reporter in 3PC mouse keratinocytes are not dramatic.*

- All referees criticized the use of mouse keratinocyte cell line 3PC. For the revised manuscript we used human keratinocytes (HaCaT). In HaCaTs, REDD1 inhibition significantly (more than two-folds) decreased GRE.Luc induction (new Fig. 7), in agreement with the *in vivo* results in REDD1 KO mice.

- ***Other concerns:*** We incorporated all suggestions changes in the text, and added the reference on autophagy (Molitoris et al., JBC, 2011). We are limited in our ability to discuss "druggability" of REDD1 by MTA with Quark Pharmaceuticals that provided REDD1 KO mice for our studies.

Referee 2.

- *Manuscript needs editorial work.*

- We extensively edited the manuscript to provide clear "story line" focused on the novel clinically important aspects of GR regulation by its target gene REDD1, and REDD1 atrophogenic role in skin.

- *Figure 6 represents the truly novel aspects of the work. Unfortunately, the results are not shared with research community.*

- Please see above. The arrays are posted on NCBI web site.

- *It is not clear how to reconcile the transient effects of glucocorticoid treatment on REDD1 protein, mTORC1 signaling, and autophagy with the sustained tissue loss.*

- In additional experiments, REDD1 induction occurred at mRNA and protein levels after each FA application albeit it was dampened at end of chronic treatment (Current Figs. 1 and 2, and pages 5-6).

- *Rationalize the choice of P-rpS6 and mTOR (Ser 2448 - an AKT site) as biomarkers of mTORC activation rather than the more typical markers such as p70S6K (Thr 389) and mTOR (Ser 2481). Concern is that rpS6 is a target for other signaling pathways.*

- Typically, mTOR activity in the skin is measured by phosphorylation of 4E-BP1 and S6K1; however rpS6, a substrate of S6K1, is also used (Checkley et al., 2011) and is quite sensitive to rapamycin. We used 4E-BP1 and rpS6 because S6K1 phosphorylation was very low in B6D2 mice even at baseline. The results for 4E-BP1 are shown in Figs. 2B and 5C. mTOR phosphorylation at Ser 2448 is mediated by S6K1 and also Akt, and was also sensitive to rapamycin (Checkley et al., 2011).

- *Concerns about value of Figure 3, and results related to miR-221.*

-Following the referee's concern, we omitted former Fig. 3 showing the effect of REDD1 on keratinocyte size.

- Please see response to Referee 1 above.

- *Are glucocorticoid treatment groups in Fig 4B significantly different?*

- We added statistical analysis, and showed that the resistance of REDD1 KO mice to epidermal hypoplasia is statistically significant (current Fig. 3C).

- *Former Figure 6 represents the novel findings reported in the manuscript. Authors should present all the gene expression data.*

- We answered this question above.

Referee #3

- *The quality of representative images is not convincing. Quantification of BrdU is necessary in Figure 4C.*

- We improved quality of photographs of glucocorticoid –treated human skin (current Fig. 1E). The photographs and morphometric analysis (Fig. 1F) clearly show the significant ~ two-fold decrease in epidermal thickness after chronic treatment of volunteers with glucocorticoid CBP.

- In former Fig. 4D (current Fig. 4A) we presented p63 (not BrdU) immunostaining. We performed the quantification of p63 (current Fig. 4B) which confirmed the protection of REDD1 KO p63+ progenitors from glucocorticoids.

- *Showing FACS data for quantification of CD34 cells would be more convincing than CD34 staining.*

- We have significant experience with FACS analysis to study stem cells in skin (Chebotaev et al., 2007a). Unfortunately, the isolation of keratinocytes by FACS from extremely thin epidermis appeared to be technically impossible. Instead, we quantified the number of CD34+ hair follicles: only 1 out of 25 hair follicles in w.t. skin contained CD34+ cells, compared to 10/25 hair follicles in REDD1 KO skin (p. 7).

- *Based on data in Fig 1D1 scraping method does not show adequate purity of fat fraction (contamination with epidermal markers (K5 and involucrin) is visible on Western blot).*

- We respectfully disagree: the analysis of keratinocyte/adipocyte markers by PCR (it was PCR analysis not Western blotting) shows ~ 95% enrichment (Supplemental Fig. 1).

- *Choice of 3PC mouse keratinocyte cell line instead of primary mouse and human keratinocytes are not justifiable and could be misleading.*

- We followed this advice, and used 3-D ORC of human epidermis made from primary human epidermal keratinocytes in which glucocorticoid CBP induced REDD1 (Fig. 5C) when we reduced the amount of hydrocortisone in the medium (page 14). As glucocorticoids did not induce REDD1 in monolayer NHEK cultures (possibly due to the high levels of glucocorticoids used in primary keratinocyte growth medium formulations such as Epilife (Cascade Biologicals), we used HaCaT human keratinocytes to assess REDD1 effect on GR phosphorylation and nuclear import.

- *Quantification of PCR is questionable – Replaced by Q-PCR*

- *REDD1-mediated atrophy by glucocorticoids has been reported in muscle tissue.*

- Please see our enumeration of the novelty of the studies above (general comments).

- *Clinical impact is unclear since the effects on human skin differ from mouse.*

© EMBO

- We answered this question above.

- *Although interesting topic that may potentially have clinical impact in the future, the quality of data, experimental design and methods limit overall enthusiasm.*

- We followed referees' comments, improved the quality of immunostaining and micro-photographs, and added significant number of new experiments/models (please see above).

- *Glucocorticoid action is not fully addressed at several levels. Confirmation of GR activation was not assessed, and levels of phosphorylated GR was not measured.*

-We evaluated the impact of REDD1 knockdown onGR activity in Luciferase assay, and several major steps in the GR activation: GR-Ser211 phosphorylation, and GR nuclear import (new Fig. 7) in human keratinocytes HaCaTs.

- *The distinction between non-genomic and genomic effects of glucocorticoids is necessary.*

- We agree with reviewer, and consider these experiments for our future studies. However, GR interacts with numerous signaling pathways via direct protein-protein interactions including Akt, MAPK, IGF, STATs. Thus, this is a widely focused research that will require significant time and effort.

- *The endogenous cortisol activity and enzymes that regulate it (HSD11b1/2) were not acknowledged and can have important influence on the data outcome.*

- The effects of glucocorticoids in REDD1 KO animals have never been studied, and the effect of REDD1 on local glucocorticoid synthesis, and the level of steroids in serum are not known. However, we found that the lack of REDD1 did not significantly affect gene expression in control adult skin (Supplemental Table 1) suggesting that there are no major differences in the level of endogenous steroids and GR activity in untreated skin of REDD1 KO mice.

- *Microarray analyses that claims 50% difference is not documented.*

- We responded to this comment above.

- *Skin atrophy includes major effects on dermal component which was not included.*

- Following referee's comment, we performed Masson's trichrome staining (current Fig. 3), and found that both collagen fiber network and dermal cellularity were protected against glucocorticoid effects in REDD1 KOs.

3rd Editorial Decision 26 September 2014

Thank you for the submission of your revised manuscript to EMBO Molecular Medicine. We have now heard back from the three Reviewers whom we asked to evaluate your manuscript.

You will see that while the Reviewers 1 and 3 are supportive of you work, Reviewer 2 points to a few issues that remain to be acted upon before we can accept your manuscript for publication.

Specifically, Reviewer 2 would like you to provide better documentation of inflammation by using additional, more accurate methods. S/he would also like you to improve the presentation quality by providing higher magnification images for Figure 3 panel A. This reviewer also mentions other issues that require you action.

In conclusion, while publication of the paper cannot be considered at this stage, we would be

pleased to consider a substantially revised submission, with the understanding that Reviewer 2's concerns must be addressed with additional experimental data where appropriate and that acceptance of the manuscript will entail a second round of review.

Please note that it is EMBO Molecular Medicine policy to allow a single round of revision only and that, therefore, acceptance or rejection of the manuscript will depend on the completeness of your responses included in the next, final version of the manuscript. EMBO Molecular Medicine now requires a complete author checklist (http://embomolmed.embopress.org/authorguide#editorial3) to be submitted with all revised manuscripts. I am attaching a copy of the checklist to this letter for your convenience.

In the event of acceptance, you will be asked to fulfil a number of editorial requirements as listed below. I suggest that you provide the following information and amendments requested with the next, final version of your manuscript:

1) As per our Author Guidelines, the description of all reported data that includes statistical testing must state the name of the statistical test used to generate error bars and P values, the number (n) of independent experiments underlying each data point (not replicate measures of one sample), and the actual P value for each test (not merely 'significant' or 'P < 0.05').
2) The manuscript must include a statement in the Materials and Methods identifying the institutional and/or licensing committee approving the experiments, including any relevant details (like how many animals were used, of which gender, at what age, which strains, if genetically modified, on which background, housing details, etc). We encourage authors to follow the ARRIVE guidelines for reporting studies involving animals. Please see the EQUATOR website for details: http://www.equator-network.org/reporting-guidelines/improving-bioscience-research-reporting-the-arrive-guidelines-for-reporting-animal-research/. Please make sure that all the above details are reported
3) We are now encouraging the publication of source data, particularly for electrophoretic gels and blots, with the aim of making primary data more accessible and transparent to the reader. Would you be willing to provide a PDF file per figure that contains the original, uncropped and unprocessed scans of all or at least the key gels used in the manuscript? The PDF files should be labeled with the appropriate figure/panel number, and should have molecular weight markers; further annotation may be useful but is not essential. The PDF files will be published online with the article as supplementary "Source Data" files. If you have any questions regarding this just contact me.
4) Every published paper now includes a 'Synopsis' to further enhance discoverability. Synopses are displayed on the journal webpage and are freely accessible to all readers. They include a short standfirst (to be written by the editor) as well as 2-5 one sentence bullet points that summarise the paper (to be written by the author). Please provide the short list of bullet points that summarise the key NEW findings. The bullet points should be designed to be complementary to the abstract - i.e. not repeat the same text. We encourage inclusion of key acronyms and quantitative information. Please use the passive voice. Please attach these in a separate file or send them by email, we will incorporate them accordingly.
6) I note that the quality of some images especially of the blots is not ideal. The resolution appears low and the bands often appear blocky/blurry when magnifying. Please use better images as these issues could lead to problems when the production team tries to resize these images for the final manuscript.

I look forward to seeing a revised form of your manuscript as soon as possible.

***** Reviewer's comments *****

Referee #1 (Remarks):

The authors have made significant improvements on the overall quality of their manuscript and have addressed my concerns. In part they did this by removing Figures which perhaps impact on the novelty of the work, and suggest that the data were not very strong in the first place.
I'm not particularly keen on the way they didn't reproduce all the reviewers' comments in the rebuttal, just quoting selections of text, and it would have been nice to compare with the initial submission as tracked changes - though this is an editorial comment.

Referee #2 (Comments on Novelty/Model System):

Potential clinical impact of these fidnings are not completely clear since majority of the data are orginiating from a mouse model and transition to human use may not be so streight forward.

Referee #2 (Remarks):

The manuscript "REDD1 (regulated in development and DNA damage response) dissociates therapeutic and adverse effects of topical glucocorticoids in skin by Baida et al reports a novel functions of REDD1 pointing out its capability to differentiate and select beneficial and adverse effects of GC in skin. The authors used multiple models to demonstrate their findings, including RADD1 KO mice, human samples, human organotypic raft cultures and HaCaT cell line. The authors properly addressed most of the previous comments, resulting in greatly improved version of the manuscript. Below are the minor comments:

In Fig 3 authors show decreased dermal cellularity in FA REDD1 KO by comparison to control. However, on a low magnification shown in panel 3A it is very difficult to visualize this increased dermal cellularity. A higher magnification of the representative histology is needed and would support better the data presented in a graph (shown in Fig 3D).

Assessment of inflammation needs to be documented by more accurate methods (histology, immunohistochemistry or flow cytometry) in addition to ear punch weight measurements (Fig 3E).

Consistency in labeling panels is important. Please relabel the Western blot panels in Fig 2B by placing a legend showing time points on a top (similar to Fig 3C).

In Fig 2C please highlight Belcin-1 by arrow and explain multiple bands in the Western blot for this protein.

Referee #3 (Comments on Novelty/Model System):

Results illustrate the role of REDD1 in the dissociation of the therapeutic and adverse effects of glucocorticoids.

Referee #3 (Remarks):

The author's responses to the previous review are adequate.

1st Revision - authors' response 16 October 2014

Response to the specific comments of reviewer # 2.

- *"Potential clinical impact of these findings are not completely clear since majority of the data are originating from a mouse model"*.

  The clinical relevance of our manuscript was demonstrated by our findings that REDD1 expression was strongly induced by the glucocorticoid CBP (we used clinically relevant topical regimen of CBP treatment of human volunteers) in human skin. We also used the best available human skin model - organotypic raft cultures made from human primary keratinocytes with blocked REDD1 expression to causatively assess the role of REDD1 in human epidermal atrophy.

- *"In Fig 3 authors show decreased dermal cellularity in FA REDD1 KO by comparison to control. However, on a low magnification shown in panel 3A it is very difficult to visualize this increased dermal cellularity. A higher magnification of the representative histology is needed".*

  As requested, we added higher magnification micro-photographs of dermis after the treatment with vehicle or with glucocorticoid FA (Supplemental Fig. 6). The text was changed accordingly (p. 7, paragraph 1).

- *"Assessment of inflammation needs to be documented by more accurate methods (histology, immunohistochemistry or flow cytometry) in addition to ear punch weight measurements".*

  As requested by reviewer, we performed the additional ear edema experiment in REDD1 KO and isogenic w.t. mice, and added the morphology of ears (H&E staining) after croton oil +/- FA treatments (New Fig. 5, and text changes on p. 7, paragraph 3).

- *"Consistency in labeling panels is important. Please re-label the Western blot panels in Fig 2B".*

  The labeling of Western blot panels in Fig. 2B was changed as suggested to maintain consistency in labeling in the Figures.

- *"Please highlight Belcin-1 by arrow and explain multiple bands in the Western blot for this protein".*

  There are indeed multiple bands on the Western blot of Beclin -1 (Fig. 2 C). In our work we used polyclonal Beclin-1 antibody #3738 from Cell Signaling that picks several bands on Western blots when proteins of mouse or human origin are used (see the Figure from Cell Signaling Ab information sheet, http://www.cellsignal.com/products/primary-antibodies/3738?Ntt=beclin&fromPage=plp) possibly reflecting Beclin 1 phosphorylation pattern. We added the information about this Ab to Materials and Methods ("Western blot analysis" section).

4th Editorial Decision 28 October 2014

Thank you for the submission of your revised manuscript to EMBO Molecular Medicine. We have now received the enclosed report from the Reviewer who were asked to re-assess it. As you will see the Reviewer is now supportive and I am pleased to inform you that we will be able to accept your manuscript. We have also recieved your completed check list.

However, I am afraid that before we can formally accept you manuscript I need one final amendment, which is a pity as I had asked you for the very same correction in my previous decision letter: As per our Author Guidelines, the description of all reported data that includes statistical testing must state the name of the statistical test used to generate error bars and P values, the number (n) of independent experiments underlying each data point (not replicate measures of one sample), and the actual P value for each test (not merely 'significant' or 'P < 0.05').

I had also informed you that we are now encouraging the publication of source data, particularly for electrophoretic gels and blots, with the aim of making primary data more accessible and transparent to the reader. Would you be willing to provide a PDF file per figure that contains the original, uncropped and unprocessed scans of all or at least the key gels used in the manuscript? The PDF files should be labeled with the appropriate figure/panel number, and should have molecular weight

markers; further annotation may be useful but is not essential. The PDF files will be published online with the article as supplementary "Source Data" files. If you have any questions regarding this just contact me. Do consider however, that this is not complusory at this time.

Finally, I would like to propose the following modified title for your manuscript: "REDD1 functions at the crossroads between the therapeutic and adverse effects of topical glucocorticoids" Would this be agreeable?

Please submit your revised manuscript within two weeks and in any case as soon as possible.

I look forward to reading a new revised version of your manuscript.

***** Reviewer's comments *****

The manuscript is suitable for publication
